# Nitrogen-cycling microbial communities respond differently to nitrogen addition under two contrasting grassland soil types

**DOI:** 10.3389/fmicb.2024.1290248

**Published:** 2024-05-30

**Authors:** Baihui Ren, Xinwei Ma, Daiyan Li, Long Bai, Jiahuan Li, Jianxin Yu, Meng Meng, Haoyan Li

**Affiliations:** College of Horticulture, Shenyang Agricultural University, Shenyang, China

**Keywords:** nitrogen addition, nitrogen cycling, source and amount of nitrogen application, grassland type, microbial community

## Abstract

**Introduction:**

The impact of nitrogen (N) deposition on the soil N-transforming process in grasslands necessitates further investigation into how N input influences the structural composition and diversity of soil N-cycling microbial communities across different grassland types.

**Methods:**

In this study, we selected two types of grassland soils in northwest Liaoning, temperate steppe and warm-temperate shrub, and conducted short-term N addition experiments using organic N, ammonium N, and nitrate N as sources with three concentration gradients to simulate N deposition. Illumina MiSeq sequencing technology was employed to sequence genes associated with N-cycling microbes including N-fixing, ammonia-oxidizing and denitrifying bacteria, and ammonia-oxidizing archaea.

**Results and discussion:**

The results revealed significant alterations in the structural composition and diversity of the N-cycling microbial community due to N addition, but the response of soil microorganisms varied inconsistent among different grassland types. Ammonium transformation rates had a greater impact on soils from temperate steppes while nitrification rates were more influential for soils from warm-temperate shrubs. Furthermore, the influence of the type of N source on soil N-cycling microorganisms outweighed that of its quantity applied. The ammonium type of nitrogen source is considered the most influential driving factor affecting changes in the structure of the microbial community involved in nitrogen transformation, while the amount of low nitrogen applied primarily determines the composition of soil bacterial communities engaged in nitrogen fixation and nitrification. Different groups of N-cycling microorganisms exhibited distinct responses to varying levels of nitrogen addition with a positive correlation observed between their composition, diversity, and environmental factors examined. Overall findings suggest that short-term nitrogen deposition may sustain dominant processes such as soil-N fixation within grasslands over an extended period without causing significant negative effects on northwestern Liaoning’s grassland ecosystems within the next decade.

## 1 Introduction

Atmospheric nitrogen (N) deposition is the process through which N precipitates onto terrestrial and marine environments in various forms as a consequence of anthropogenic activities (Du et al., 2014), such as fossil fuel combustion and industrial emissions. One of the primary drivers behind the global decline in terrestrial biodiversity is N deposition (Clark et al., 2013), with numerous studies demonstrating that the frequent addition of N leads to a rapid decline in the diversity of aboveground species (Fang et al., 2012), soil acidification (Tian and Niu, 2015), and alterations in ecosystem functioning (Greaver et al., 2016). According to the analysis based on the published N deposition data, the national average atmospheric N deposition has increased from 11.11 to 13.87 kg ha^−1^ yr^−1^ over 30 years, an increase of approximately 25% (Jia et al., 2016). Grasslands, one of the most prevalent vegetation types globally, cover approximately one-third of the earth’s land area and play a crucial role in terrestrial ecosystems (Kemp et al., 2013; Merbold et al., 2014). China is a vast country abundant in grassland resources, with the grassland area that accounts for approximately 41.7% of the total land (Shen et al., 2015). Consequently, the impact of N deposition on grassland ecosystems has become a matter of significant concern (Lü et al., 2019). The ecological impacts of N deposition can exhibit significant variations depending on the type of grassland (Qi et al., 2021). Northwest Liaoning, located in central Northeast Asia, possesses abundant grassland resources, primarily utilized for grazing purposes. This region experiences a semi-arid monsoon continental climate characterized by substantial annual and daily temperature fluctuations. Due to inadequate soil management practices and water scarcity issues persisting over the years, the soils demonstrate heightened sensitivity to external environmental changes (Peel et al., 2007; Tong, 2012).

The grassland soil N cycle plays a crucial role in grassland ecosystems, and N deposition significantly affects soil N transformation processes in grasslands (Jiang et al., 2018). Soil microorganisms are important drivers of nutrient cycling and energy flow in ecosystems (Hallin et al., 2009). Previous studies have demonstrated that soil N-cycling microorganisms played a key role in the soil N cycle and influenced the N-cycling processes by participating in N fixation, mineralization, nitrification, denitrification, and anaerobic ammonium oxidation within grassland ecosystems (Harter et al., 2014). For instance, biological N fixation, predominantly mediated by soil N-fixing bacteria, is widely acknowledged as the primary source of N input in grasslands (Cleveland et al., 1999), whereas nitrification processes are attributed to ammonia-oxidizing microorganisms (Jia and Conrad, 2009). Denitrification processes largely depend on denitrifying microorganisms for nitrate or nitrite reduction (Zumft, 1997). The phenomenon of N deposition, which directly affects the soil N cycle, disrupts the indigenous soil microbial community. Studies have shown that the structural composition and diversity of soil microbial communities exhibited distinct responses depending on both the type and quantity of the applied N source (Dambreville et al., 2006; Ren et al., 2022). For example, the abundance of ammonia-oxidizing bacteria increased with nitrate-N fertilization (Wang et al., 2019), and the inorganic N application altered denitrifying bacterial community structure while organic N had no effect on it (Yin et al., 2014). Soil microorganisms exhibit high sensitivity to changes in environmental conditions, leading to alterations in their community structure and composition for rapid adaptation to environmental shifts, which may result in limitations in certain microbial communities (Victoria et al., 2021; Wang et al., 2021). For example, high pH environments exerted a positive influence on ammonia-oxidizing bacteria while negatively impacting ammonia-oxidizing archaea (Ying et al., 2017). In high-N environments, the abundance of most N-converting functional genes increased, whereas the abundance of *nifH* genes significantly decreased (Silveira et al., 2021). In addition, there existed a significant correlation between soil N conversion rate and N-converting microorganisms, which was profoundly affected by N addition. The rates of soil ammonification, nitrification, and N mineralization are regulated by N-fixing bacteria, ammonia-oxidizing archaea, ammonia-oxidizing bacteria, and denitrifying bacteria (Wang et al., 2017). The addition of N substantially enhanced the soil net N mineralization rate as well as ammonium and nitrification rates (Hu et al., 2021), with changes primarily driven by variations in soil nitrate and ammonium N contents (Yang et al., 2019; Ma et al., 2021).

Although numerous studies have investigated the impact of N application on N-transforming microorganisms in grassland ecosystems, most of these studies have primarily focused on gene copy number levels, with limited attention given to their diversity and structure. In this study, we employed high-throughput sequencing technology to analyze the taxonomic composition and structure characteristics of soil N-transforming microbial communities (N-fixing bacteria, ammonia-oxidizing archaea, ammonia-oxidizing bacteria, and denitrifying bacteria) under varying N sources and levels in two types of grassland soils located in northwestern Liaoning province. Additionally, we integrated these findings with the corresponding environmental factor data to elucidate the influence of N addition on soil microbial communities involved in N transformation in different grassland types. Overall, we proposed three hypotheses: (1) The structural composition and diversity of N-transforming microbial communities in different grassland types in northwest Liaoning are significantly influenced by the source and application amount of N. (2) The response of soils from different grassland types to N addition varies. (3) Any alteration in environmental factors may impose limitations on N-transforming microorganisms.

## 2 Materials and methods

### 2.1 Experimental design

The experiment was conducted at the experimental base of Shenyang Agricultural University (41°50′N, 123°34′E). Two types of grassland soils, temperate steppe and warm-temperate shrub, were utilized as a cultivation substrate for the pot test (1.5 kg/pot). The basic information of soil for each grassland type is shown in Table 1. With a test period of 90 days, ice grass (*Agropyron cristatum* (L.) Gaertn.) was cultivated as the dominant species in the temperate steppe, while long manzanita (*Stipa bungeana* Trin.) served as the dominant species in the warm-temperate shrub.

**Table 1:** Basic information regarding soils of different grassland types.

Group	TS^a^	WST^b^
Latitude and Longitude	N 42°48ˊ, E122°32ˊ	N 42°3ˊ, E 120°5ˊ
TN/(g∙kg^−1^)^c^	0.12 ± 0.01	0.87 ± 0.01
TP/(g∙kg^−1^)^d^	0.31 ± 0.02	0.61 ± 0.03
SOC/(g∙kg^−1^)^e^	1.20 ± 0.14	8.07 ± 0.52
pH	5.74 ± 0.05	7.07 ± 0.12
EC/(S∙m^−1^)^f^	44.85 ± 7.27	74.75 ± 18.69
AN/(mg∙kg^−1^)^g^	15.17 ± 5.78	74.08 ± 8.38
NH_4_^+^-N/(mg∙kg^−1^)^h^	3.17 ± 0.72	3.10 ± 0.05
NO_3_^−^-N/(mg∙kg^−1^)^i^	5.32 ± 1.26	9.93 ± 2.28
SP/(mg∙kg^−1^)^j^	21.39 ± 1.61	2.81 ± 0.79
ON/(mg∙kg^−1^)^k^	0.12 ± 0.01	0.87 ± 0.01

a TS represents temperate steppe.

b WST represents warm-temperate shrub.

c TN represents total nitrogen.

d TP represents total phosphorus.

e SOC represents soil organic carbon.

f EC represents electrical conductivity.

g AN represents alkali-hydrolyzable nitrogen.

h NH_4_^+^-N represents ammonium nitrogen.

i NO_3_^−^-N represents nitrate nitrogen.

j SP represents available phosphorus.

k ON represents organic nitrogen.

The same as below.

The three types of nitrogen used were organic N (C_2_H_5_NO_2_), ammonium N (NH_4_Cl), and nitrate N (Ca(NO_3_)_2’_4H_2_O), with concentration gradients of 0.15 g/kg, 0.30 g/kg, and 0.45 g/kg, respectively (the N applied was pure N). Each gradient of each N fertilizer was replicated four times, and the control group for each grass type had four replicates as well, resulting in a total of 80 pots–40 pots per grass type. The schematic diagram of nitrogen application is shown in Supplementary Figure S1.

### 2.2 Sample collection and determination

Soil samples were collected on the 10th, 30th, 60th, and 90th days of incubation and were divided into two portions. One portion was stored at 4°C for physicochemical property analyses (soil organic carbon, pH, conductivity, total phosphorus, fast-acting phosphorus, total soil nitrogen, alkaline soluble nitrogen, ammonium nitrogen, nitrate nitrogen, and organic nitrogen) according to Bao (2000). The 90th day remaining soil samples were frozen at −80°C for high-throughput sequencing analyses. The calculation formula for soil nitrogen conversion rate is as follows (Widdig et al., 2020):

Soil net nitrogen mineralization rate mg·kg^−1^·d^−1^ = [after cultivation (NH_4_^+^-N + NO_3_^−^-N) – before cultivation (NH_4_^+^-N + NO_3_^−^-N)]/day,

Soil net nitrogen nitrification rate mg·kg^−1^·d^−1^ = [NO_3_^−^-N after cultivation - NO_3_^−^-N before cultivation]/day,

Soil net nitrogen ammonification rate mg·kg^−1^·d^−1^ = [NH_4_^+^-N after cultivation - NH_4_^+^-N before cultivation]/day.

### 2.3 Illumina sequencing

Four typical N-cycling microbial communities were investigated in this study: N-fixing bacteria (*nifH*) of nitrogen fixation, ammonia-oxidizing archaea (AOA), ammonia-oxidizing bacteria (AOB) with ammonia monooxygenase genes (*amoA*), and denitrifying bacteria (*nirK*) of nitrite reduction (Yin et al., 2014).

Genomic DNA extraction: The Power Soil DNA Isolation Kit (MoBio, United States) was used to extract genomic DNA from the samples, after which the purity and concentration of the DNA were tested using agarose gel electrophoresis and Nanodrop analysis.

PCR amplification: The diluted genomic DNA was used as a template for the polymerase chain reaction (PCR) using specific primers with barcodes and efficient high-fidelity enzymes according to the selection of sequencing regions. Detailed information about the primers and the PCR protocols for the four N-cycling microbial communities could be found in Supplementary Table S1.

High-throughput sequencing library was constructed using the TruSeq^®^ DNA PCR-Free Sample Preparation Kit. The libraries were quantified using Qubit and qPCR. After the libraries were quantified, the v2 sequencing kit (2 × 250 bp) and MiSeq sequencer were used for onboard sequencing at Shanghai Personal Biotechnology Co., Ltd.

### 2.4 Processing of sequencing data

The initial sequence was acquired using the Quantitative Insights into Microbial Ecology (QIIME v.1.9.1) quality control process to extract high-quality clean tags (Bolyen et al., 2018),^1^ and the sequences were clustered into operational taxonomic units according to 97% pairwise identity using Vsearch (v2.13.4). RDP FrameBot^2^ was used to correct sequencing errors (insertions & deletions) and translate DNA sequences for functional genes. The sequence database referenced in this study was obtained from the National Center for Biotechnology Information (NCBI). Alpha diversity metrics (Chao, 1984), observed species, (Shannon, 1948; Simpson, 1949), Pielou’s evenness (Pielou, 1966), and Good’s coverage (Good, 1953) and beta diversity metrics (Bray–Curtis dissimilarity) were estimated using the diversity plugin with samples rarefied to sequences.

The DNA sequences used in this study were obtained from the National Center for Biotechnology Information Sequence Read Archive database under the accession number PRJNA929669.

### 2.5 Statistical analyses

One-way analysis of variance (ANOVA) was conducted using SPSS software (version 22.0, IBM Corporation, Armonk, NY, United States) to determine the differences in environmental factors and the abundance of soil N-transforming microorganisms among the treatments. Two-factor and multi-factor ANOVAs were conducted on N application, N source, and incubation time to determine whether their interactions had an effect on the study. The Pearson correlation analysis was conducted to explore the correlation between environmental factors and soil N-transformed microbial abundance. Additionally, the analysis examined the correlation with alpha diversity. Significant differences between treatments were confirmed using Tukey’s HSD test at *p* < 0.05.

Box plots were drawn using the “ggplot2” package in R, and the compositional distribution of each sample was visualized at the phylotaxonomic genus level to determine differences in the N-converted microbial community composition and alpha diversity in different grassland soil types. The structural composition of N-transforming microbial communities in different grassland soil types was visualized using the “ape” package in R using the principal coordinate analysis based on the Bray–Curtis heterogeneity matrix. Analysis of similarities (ANOSIM) was used to investigate the effect of grassland soil types on the β-diversity of N-transforming microorganisms. In addition, the SparCC algorithm was used with the “ggraph” and “igraph” packages to construct network topology index tables to observe differences in different N-cycling microbial communities. Finally, the OmicShare tool was used to produce a network heat map of the environmental factors and the composition and diversity of the N-transforming microbial community.

## 3 Results

### 3.1 Nitrogen addition significantly affected soil physicochemical properties in grasslands

As presented in Supplementary Table S2, the application of N resulted in an increase in soil total N, conductivity, alkaline N, ammonium N, nitrate N, and organic N. The magnitude of this effect was positively correlated with the amount of applied N and was most pronounced for ammonium N fertilization. However, no significant changes were observed in soil total P and fast-acting P contents following the application of N; instead, a decrease in the soil total P content was noted. The application of N significantly increased the soil organic carbon content (*p* < 0.05) in the temperate steppe but not in the warm-temperate shrub. Furthermore, while there were no significant effects on soil pH levels following the addition of nitrogen to the temperate steppe, it did have a negative impact on pH levels within the warm-temperate shrub, especially when using ammonium N fertilizers (*p* < 0.05). Additionally, the sampling time significantly affected soil total N, total P, pH, ammonium N, fast-acting P, and organic N (*p* < 0.05), showing an increasing trend followed by the decreasing trend peaking at 30 days. In the temperate steppe, significant or highly significant differences were observed in soil total P, organic carbon, and fast-acting P contents at different incubation times (*p* < 0.05 or *p* < 0.01). In the warm-temperate shrub, there were significant or highly significant differences in soil total N, pH, ammonium N, fast-acting P, and organic N contents (*p* < 0.05 or *p* < 0.01). In addition, with the exception of the soil ammonium N content, significant or highly significant differences (*p* < 0.05, *p* < 0.01) were found in the measured basic physicochemical properties of the different grassland types.

### 3.2 Nitrogen addition significantly affected the soil N conversion rate in grasslands

**Figure 1:**
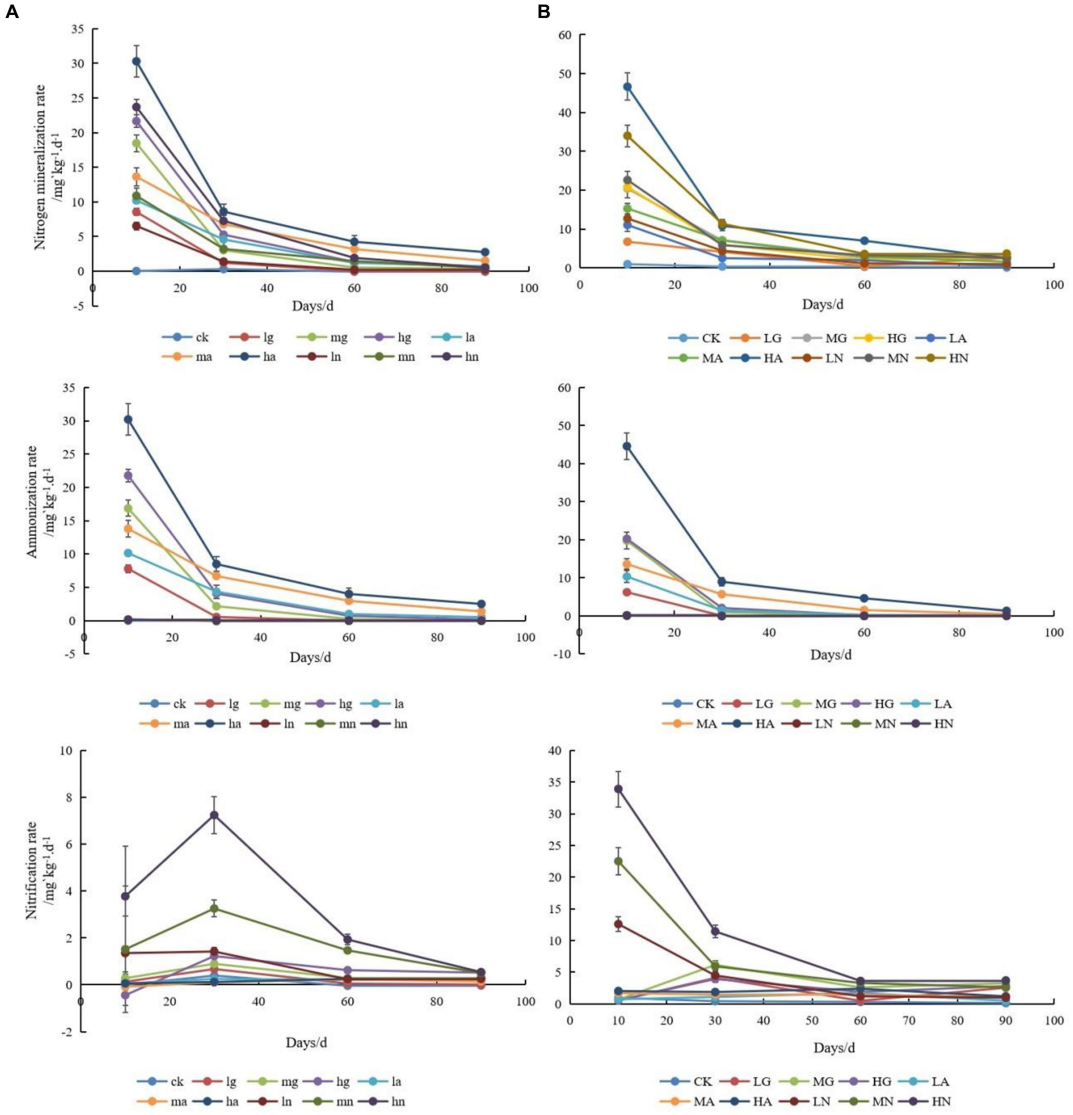
Changes in soil N-cycling rates of temperate steppe **(A)** and warm-temperate shrub **(B)** grasslands under different treatments at different times. ck, control; lg, low amount of organic N addition; mg, medium amount of organic N addition; hg, high amount of organic N addition; la, low amount of ammonium N addition; ma, medium amount of ammonium N addition; ha, high amount of ammonium N addition; ln, low amount of nitrate N addition; mn, medium amount of nitrate N addition; hn, high amount of nitrate N addition; the lowercase is used to represent temperate steppe, while the uppercase is employed for warm-temperate shrub. The same as below.

The application of N significantly enhanced soil N mineralization and ammonium conversion rates (*p* < 0.05), with the highest rates observed when applying ammonium N. Moreover, increasing the N application resulted in higher soil N mineralization and ammonium conversion rates, which gradually declined with time. In addition, the rates of N mineralization and ammonification in the soils of the warm-temperate shrub were much higher than those of the temperate steppe (Figure 1).

The nitrification rates of soils differed significantly across grassland types (*p* < 0.05), with the highest rates observed in soils treated with nitrate-N source type. In the temperate steppe, the soil nitrification rate exhibited an initial increase followed by a decrease, and neither N source types nor N application amount had a significant effect on the rate (*p* > 0.05). However, it showed a time-dependent increase and ultimately demonstrated a positive correlation between the N application rate and soil nitrification rate, and the application of ammonium and nitrate N source significantly increased the soil nitrification rate (*p* < 0.05). In the warm-temperate shrub, the N source types significantly affected the soil nitrification rate (*p* < 0.05), while the nitrate N application led to an increase that gradually declined over time (*p* < 0.05). However, the application of organic N showed an increase followed by a decrease in the soil nitrification rate. The amount of applied N did not have a significant impact on the soil nitrification rate (*p* > 0.05), but its significance increased over time and eventually revealed a positive correlation between the amount of applied N and soil nitrification rate. Additionally, the nitrification rate of soils in the warm-temperate shrub was considerably higher than that observed in the temperate steppe.

### 3.3 Nitrogen addition significantly affected the composition of soil microbial communities in grasslands

**Figure 2:**
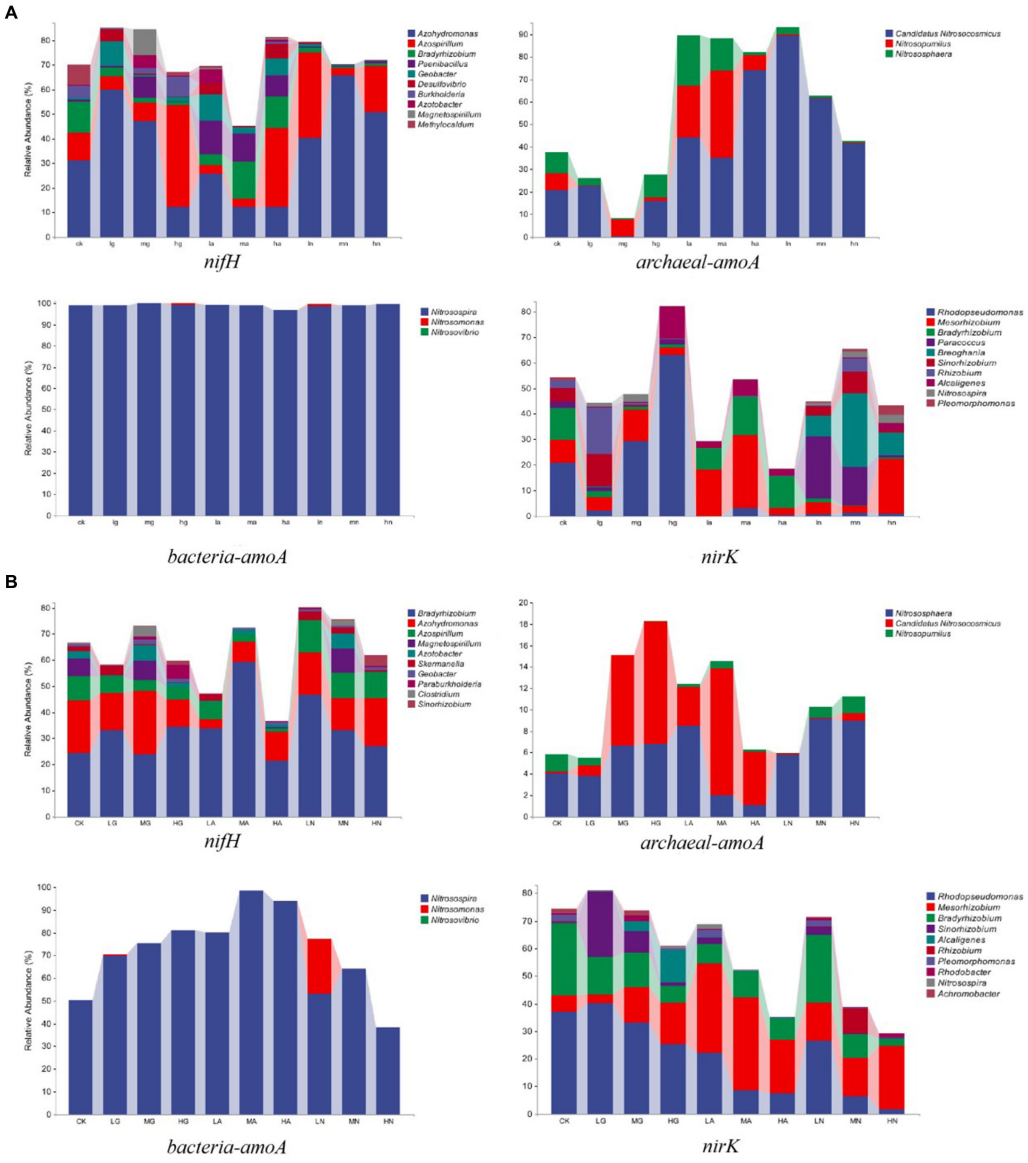
Relative abundance of soil N-cycling microbial communities at the genus level in grasslands of temperate steppe **(A)** and warm-temperate shrub **(B)**.

The N-fixing bacterial community was dominated by *Azohydromonas*, *Bradyrhizobium,* and *Azospirillum,* while the ammonia-oxidizing archaea community was mainly composed of *Candidatus Nitrosocosmicus*, *Nitrososphaera,* and *Nitrosopumilus*. *Nitrosospira*, *Nitrosomonas,* and *Nitrosovibrio* were the major genera in the ammonia-oxidizing bacterial community, while *Rhodopseudomonas*, *Mesorhizobium,* and *Bradyrhizobium* were the major genera in the denitrifying bacterial community. Notably, N addition significantly influenced the relative abundance of different genera (*p* < 0.05), with varying effects observed among different grassland types (Figure 2).

In the temperate steppe, *Azohydromonas*, *Candidatus Nitrosocosmicus*, *Nitrosospira*, and *Rhodopseudomonas* were the dominant genera in the N-fixing bacterial community, ammonia-oxidizing archaea, ammonia-oxidizing bacteria, and denitrifying bacterial communities, with mean relative abundances of 35.76, 42.02, 70.50, and 12.18%, respectively. The application of nitrate source N type significantly increased the relative abundance of *Candidatus* and *Nitrosocosmicus* (*p* < 0.05), and the organic source N type application led to a significant increase in the relative abundance of *Nitrosospira* (*p* < 0.05). Ammonium and nitrate source N type applications caused a significant decrease in the relative abundance of *Rhodopseudomonas* (*p* < 0.05), while the application of organic and nitrate source N was found to significantly reduce the relative abundance of *Bradyrhizobium* (*p* < 0.05). Furthermore, the amount of medium and high N additions resulted in a significant decrease in the relative abundance of *Nitrosovibrio* (*p* < 0.05).

In the warm-temperate shrub, *Bradyrhizobium*, *Nitrososphaera*, *Nitrosospira*, and *Rhodopseudomonas* were identified as the dominant genera in the N-fixing bacterial communities (with average relative abundances of 33.73%), ammonia-oxidizing archaeal communities (5.70%), ammonia-oxidizing bacterial communities (99.03%), and denitrifying bacterial communities (20.85%), respectively. The application of ammonium source N type significantly reduced the relative abundance of *Azospirillum* (*p* < 0.05), while both organic and ammonium source N applications significantly increased the relative abundance of *Candidatus* and *Nitrosocosmicus* (*p* < 0.05). Additionally, the application of organic source N type significantly increased the relative abundance of *Rhodopseudomonas* (*p* < 0.05). Furthermore, the amount of high N addition resulted in a significant reduction in the relative abundance of *Bradyrhizobium* (*p* < 0.05).

### 3.4 Microbial community diversity of the two grassland types under different nitrogen application treatments

**Figure 3:**
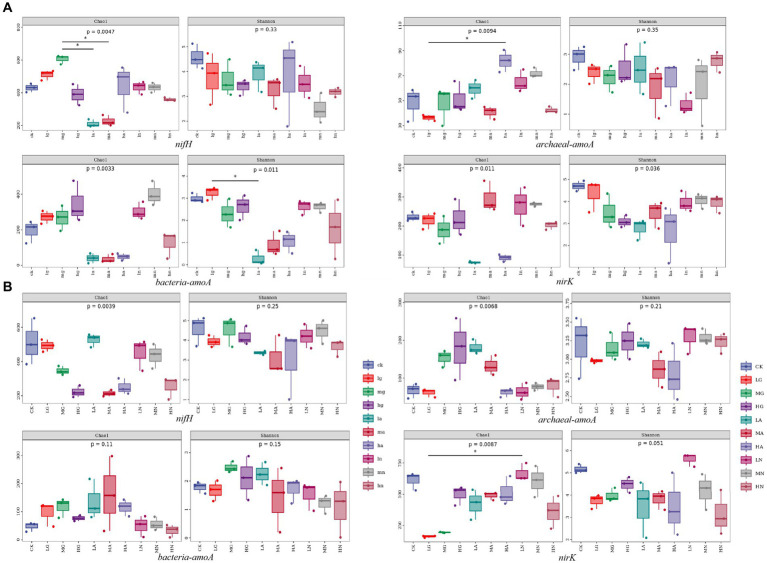
Box plot of alpha diversity grouping of soil N-cycling microbial communities in grasslands of temperate steppe **(A)** and warm-temperate shrub **(B)**.

The alpha diversity of soil N-fixing bacteria, ammonia-oxidizing archaea, ammonia-oxidizing bacteria, and denitrifying bacterial communities varied among different grassland types and was significantly influenced by different types and amounts of N application treatments (*p* < 0.05, Figure 3).

In the temperate steppe, the alpha diversity of soil N-fixing bacterial communities was significantly influenced by the N source types (*p* < 0.05), while the amount of N applied did not significantly affect their alpha diversity. The Chao1 index showed the highest value when medium organic N was applied and was significantly higher than that of other N treatments (*p* < 0.05). The application of different N sources generally reduced the alpha diversity of the N-fixing bacterial community; however, only organic N source application increased the species richness within these communities. Neither the N source nor the amount of applied N significantly affected the alpha diversity of the soil ammonia-oxidizing archaeal community. The Chao1 index was the highest with the application of high amounts of ammonium N, which was significantly higher than the other N treatments (*p* < 0.05). The sources of N types significantly affected the alpha diversity of ammonia-oxidizing bacterial communities in the grassland soils (*p* < 0.05), whereas the amount of applied N did not have a significant effect on their alpha diversity. Among them, the application of ammonium N significantly reduced the alpha diversity of ammonia-oxidizing bacterial communities (*p* < 0.05). The N source types significantly affected the alpha diversity of the denitrifying bacterial community in the grassland soils (*p* < 0.05), whereas the amount of applied N did not have a significant impact on the alpha diversity. The application of ammonium N significantly reduced the diversity of the denitrifying bacterial community (*p* < 0.05).

In contrast, in the warm-temperate shrub, the amount of N application significantly affected the alpha diversity of soil N-fixing bacterial communities in the grasslands (*p* < 0.05), while no significant effect was observed for N source types on the diversity. Almost all treatments resulted in a reduction in the alpha diversity of the N-fixing bacterial community, with high N application showing a significant decrease in species richness and exhibiting a negative correlation with the alpha diversity (*p* < 0.05). The alpha diversity of soil ammonia-oxidizing archaeal communities was not significantly affected by neither the N source nor the amount of applied N. There was no significant effect of the amount of N application on the soil ammonia-oxidizing bacterial community, but the N source types significantly affected the alpha diversity in the grasslands (*p* < 0.05). The N source types significantly affected the alpha diversity of the soil denitrifying bacterial community in the grasslands (*p* < 0.05), whereas there was no significant effect observed for the amount of N application on the alpha diversity. Organic and ammonium types of N application led to a significant reduction in the alpha diversity of the denitrifying bacterial community (*p* < 0.05).

### 3.5 Microbial community structure of the two grassland types under different nitrogen application treatments

**Figure 4:**
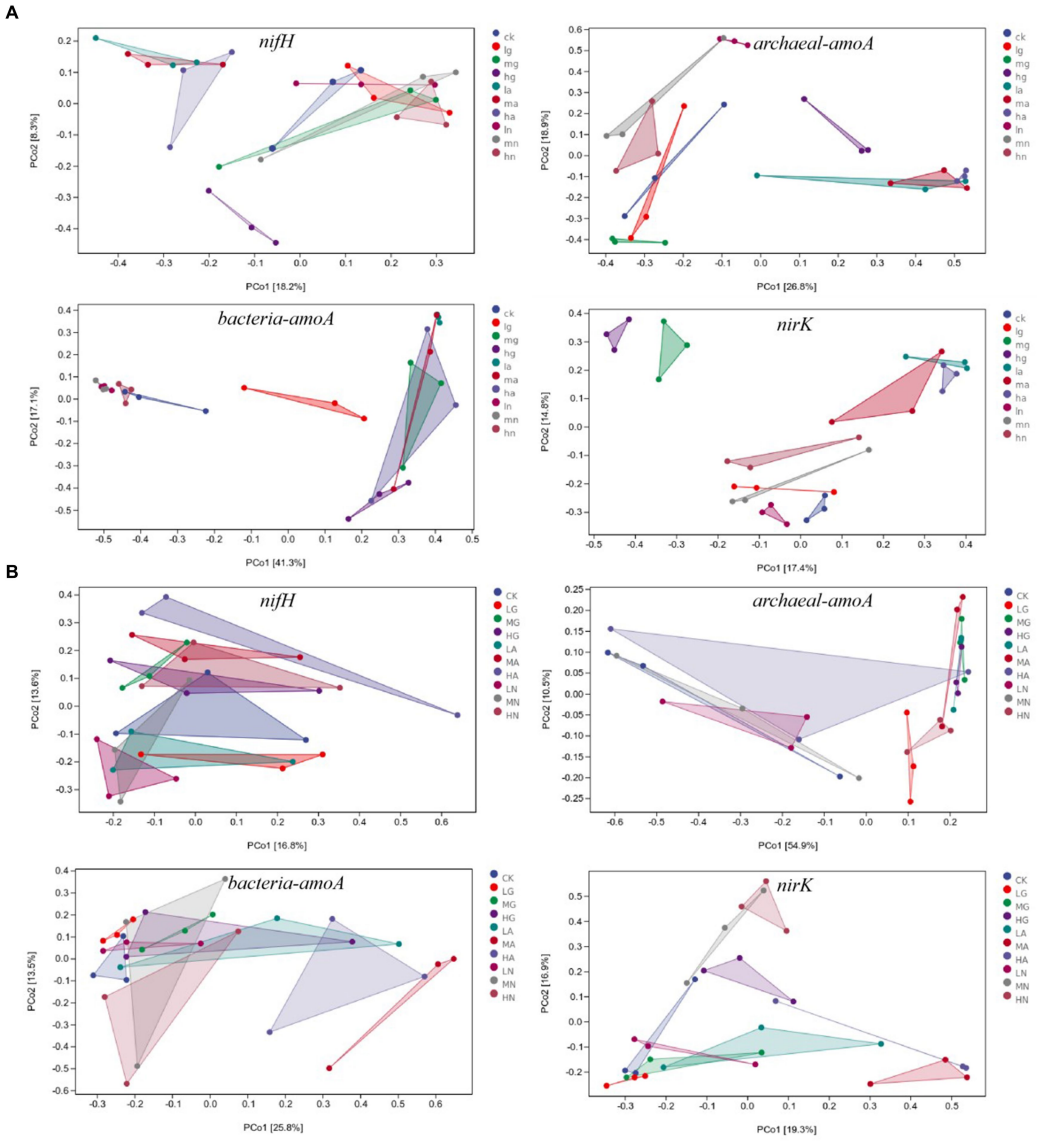
PCOA of soil N-cycling microbial communities in grasslands of temperate steppe **(A)** and warm-temperate shrub **(B)**.

The beta diversity of N-fixing bacteria, ammonia-oxidizing archaea, ammonia-oxidizing bacteria, and denitrifying bacterial communities was significantly affected by different N application treatments (*p* < 0.05). In the temperate steppe, the N source types had a significant impact on the beta diversity of N-fixing bacteria, ammonia-oxidizing archaea, ammonia-oxidizing bacteria, and denitrifying bacterial communities (*p* < 0.05), whereas the amount of N application only significantly affected the beta diversity of denitrifying bacterial communities (*p* < 0.05). In the warm-temperate shrub, N source types significantly affected the beta diversity of ammonia-oxidizing archaea, ammonia-oxidizing bacteria, and denitrifying bacteria communities (*p* < 0.05), whereas the amount of N application significantly influenced the beta diversity of denitrifying bacterial communities and N-fixing bacteria (*p* < 0.05, Figure 4).

According to the ANOSIM analysis, significant differences were observed in the beta diversity of N-fixing bacterial communities between those without N addition and those with ammonium and nitrate types of N application (*p* < 0.05), as well as between organic and ammonium types of N application in the temperate grassland soils. There were significant differences between the treatments in the beta diversity of ammonia-oxidizing archaeal bacterial communities (*p* < 0.05) with the exception between no N addition and organic N application (*p* > 0.05). Significant differences were observed in the beta diversities of ammonia-oxidizing bacterial communities among different N source types (*p* < 0.05). The beta diversity of denitrifying bacterial communities significantly differed across various N sources (*p* < 0.05), and the amount of N applications with low to medium and high N significantly affected the beta diversity of denitrifying bacterial communities (*p* < 0.05). In the warm-temperate shrub, there were significant differences between the beta diversity of N-fixing bacterial communities with low, medium, and high amounts of N applications (*p* < 0.05). Furthermore, significant differences were found in the beta diversity of ammonia-oxidizing archaeal communities between those without N addition and those with organic and ammonium types of N application, and between those with organic and nitrate types of N application (*p* < 0.05). Significant differences (*p* < 0.05) were identified in the beta diversity of ammonia-oxidizing bacterial communities between those without N addition and those with ammonium and organic types of N application, as well as between those with ammonium N and nitrate types of N. Finally, significant differences were observed in the beta diversity of denitrifying bacterial communities between those with the application of ammonium type of N and other N sources, between those with organic and nitrate types of N application, and the amount of N applications of low-to-medium and high N significantly affected beta diversity of denitrifying bacterial communities(*p* < 0.05).

### 3.6 Network characteristics of microbial communities in both grassland types under different nitrogen application treatments

The network topology parameters presented in Table 2 demonstrated that, within the temperate steppe, the symbiotic networks of N-fixing bacteria, ammonia-oxidizing archaea, ammonia-oxidizing bacteria, and denitrifying bacteria consisted of 269, 64, 92, and 122 nodes, respectively, with 834, 393, 1,689, and 2,537 edges, correspondingly. In the warm-temperate shrub, the symbiotic networks of N-fixing bacteria, ammonia-oxidizing archaea, ammonia-oxidizing bacteria, and denitrifying bacteria comprised 271, 116, 56, and 57 nodes, respectively, with 1,104, 1,382, 560, and 551 edges, correspondingly. In addition, the N-fixing bacterial community exhibited the shortest average path length t and the highest levels of the stability and assortativity across all grassland types, indicating its dominance and complexity within this study habitat.

**Table 2:** Symbiotic network topology index of soil N transforming microbial communities.

Group	*nifH*		*archaeal-amoA*		*bacterial-amoA*		*nirK*	
	TS	WST	TS	WST	TS	WST	TS	WST
Average nearest neighbor degree	8.3428	11.2565	14.9508	33.958	42.9504	21.8277	47.8966	21.8418
Average path length	7.4599	5.0908	2.4425	2.0702	1.6046	1.65	1.6792	1.7249
Degree assortativity	0.6565	0.5099	0.2447	0.0164	0.247	0.0316	0.1229	0.2607
Cluster num	9	12	1	1	1	1	1	1
Transitivity	0.6191	0.6308	0.6469	0.5207	0.6123	0.5374	0.53	0.5832
Vertice	269	271	64	116	92	56	122	57
Edge	834	1,104	393	1,382	1,689	560	2,537	551
Modularity	0.7193	0.7678	0.5434	0.3939	0.3561	0.5193	0.5136	0.3313

### 3.7 Correlations between physicochemical properties and soil nitrogen transforming microorganisms

**Figure 5:**
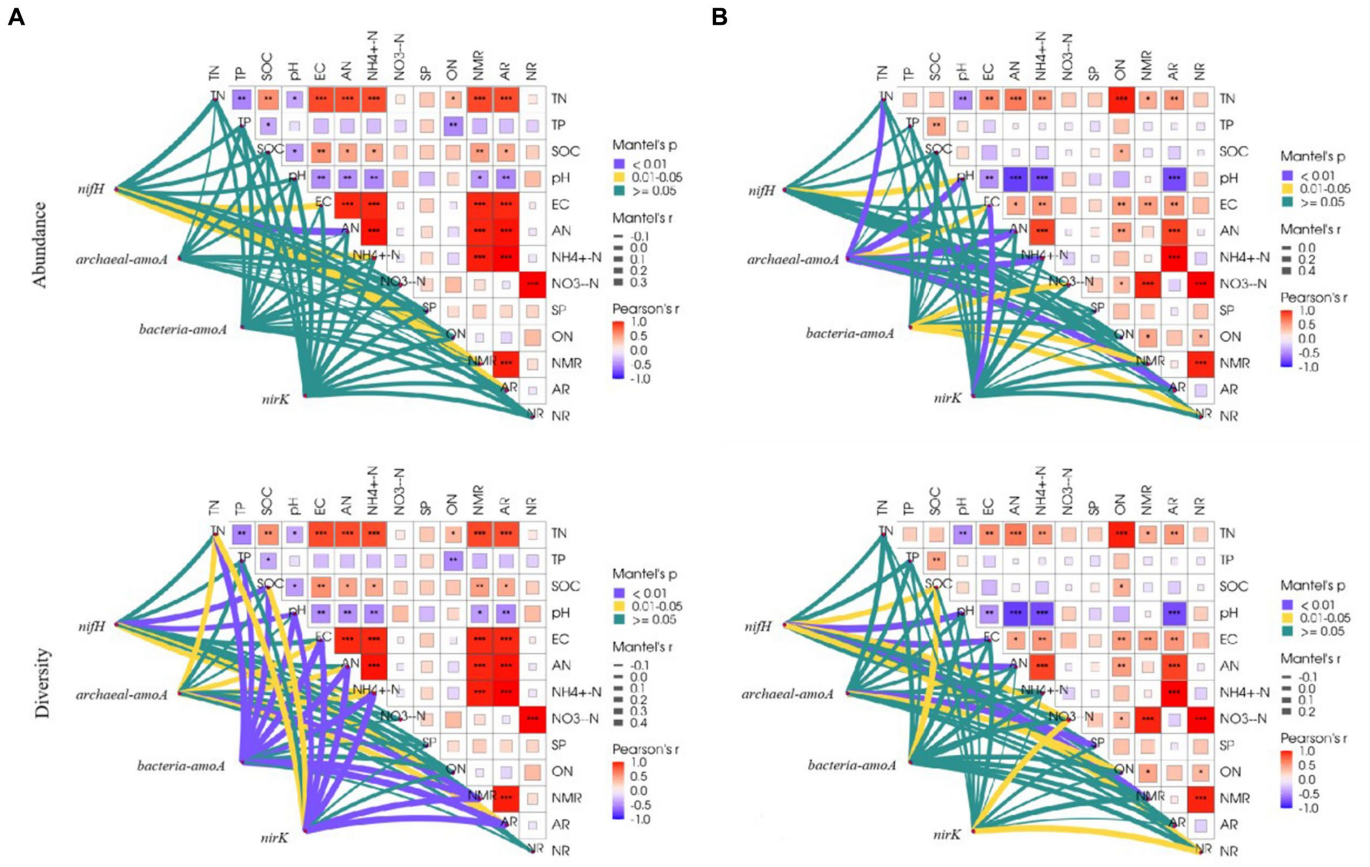
Correlation of environmental factors with species composition and alpha diversity of soil N-cycling microbial communities in grasslands of temperate steppe **(A)** and warm-temperate shrub **(B)**.

Correlations between physicochemical properties differed among the grassland types. In the temperate steppe, there was a highly significant and positive correlation between the rate of soil N mineralization and the rate of ammonium transformation (*p* < 0.01). Conversely, in the warm-temperate shrub, there was a highly significant and positive correlation between the rate of soil N mineralization and the rate of nitrification (*p* < 0.01). Additionally, in the warm-temperate shrub, there was a stronger correlation between nitrate and organic N contents compared to that observed for organic carbon content (Figure 5).

The influence of physicochemical properties varied among the grassland types. In the temperate steppe, soil conductivity, alkaline and ammonium N content, N mineralization rate, and ammonification rate were significantly or highly significantly and positively correlated (*p* < 0.05 or *p* < 0.01, respectively) with the species composition of the N-fixing bacterial community. However, no significant correlations were observed between physicochemical properties and the species composition of the other N-transforming microbial communities. Soil organic carbon, pH, ammonium N content, and ammonification rate were significantly or highly significantly and positively correlated with the alpha diversity of the N-fixing bacterial community (*p* < 0.05 or *p* < 0.01, respectively). Soil total N, conductivity, alkaline-solubilized N, ammonium N content, N mineralization rate, and ammonification rate were significantly or highly significantly and positively correlated (*p* < 0.05 or *p* < 0.01) with the alpha diversity of the ammonia-oxidizing archaeal community. Soil total N, total P, organic carbon, pH, conductivity, alkaline N, ammonium N content, ammonification rate, and nitrification rate were significantly and positively correlated with the alpha diversity of ammonia-oxidizing bacterial communities (*p* < 0.01). Soil total N, organic carbon, pH, conductivity, alkaline N, ammonium N content, ammonification rate, and nitrification rate were significantly or highly significantly and positively correlated with the alpha diversity of the denitrifying bacterial community (*p* < 0.05 or *p <* 0.01).

In the warm-temperate shrubs, soil pH was significantly and positively correlated (*p* < 0.05) with the species composition of the N-fixing bacterial community. Soil total N, pH, conductivity, alkaline N, ammonium N content, and ammonification rate were significantly or highly significantly and positively correlated (*p* < 0.05 or *p* < 0.01) with the species composition of the ammonia-oxidizing archaeal community. The soil ammonium N content, N mineralization rate, and nitrification rate were significantly and positively correlated (*p* < 0.05) with the species composition of the ammonia-oxidizing bacterial community. Soil conductivity was significantly correlated with the species composition of the denitrifying bacterial community (*p* < 0.01). Soil organic carbon, pH, conductivity, alkaline N, nitrate content, N mineralization rate, and nitrification rate were significantly or highly significantly and positively correlated with the alpha diversity of the N-fixing bacterial community (*p* < 0.05 or *p* < 0.01). Soil fast-acting P and organic N content were significantly or highly significantly and positively correlated with the alpha diversity of the ammonia-oxidizing archaeal community (*p* < 0.05 or *p* < 0.01). The soil organic carbon content was significantly and positively correlated (*p* < 0.05) with the alpha diversity of ammonia-oxidizing bacterial communities. The soil nitrate-N content and nitrification rate were significantly and positively correlated with the alpha diversity of the denitrifying bacterial community (*p* < 0.05).

## 4 Discussion

The addition of N significantly influenced the species composition of soil N-transforming microbial communities in grasslands, with varying effects observed among different grassland types. Previous studies have demonstrated that the environment factors could drive directional shifts in dominant genera of microbial communities (Sovová et al., 2020). In our study, we found distinct dominant genera of N-fixing bacteria and ammonia-oxidizing archaea across different grassland types. This variation may be attributed to the stronger soil nitrification processes occurring in warm-temperate shrubs (Figure 2). In natural ecosystems, soil nitrogen conversion is primarily driven by nitrification during the early growth seasons, while ammonium conversion becomes more prominent toward the end and non-growth seasons (Liu and Ma, 2021). Notably, the end of the growing season occurs later in warm-temperate shrubs compared with temperate steppes (Lee et al., 2002; Hu et al., 2014). In this study, the rate of soil N mineralization in the temperate steppe was significantly and positively correlated with the ammonification rate, while it showed a stronger positive correlation with the nitrification rate in the warm-temperate shrub, demonstrating that the warm-temperate shrub had a stronger nitrification process than the temperate steppe (*p* < 0.01, Figure 5). In addition, *Azohydromonas* cannot survive anaerobically in environments containing nitrate and nitrite (Nguyen and Kim, 2017), leading to decreased viability and relative abundance in areas with high levels of nitrification. Ammonia-oxidizing archaea primarily derive energy from the oxidation of ammonia, a process that necessitates the presence of oxygen. Nevertheless, certain archaea, including *Nitrososphaera* (Lu and Jia, 2013; Wang et al., 2014), have been observed to thrive in anoxic environments (Kraft et al., 2022). Conversely, warm-temperate shrub-like soils exhibited lower levels of oxygenation (Cook et al., 2013), which adversely impacted genera other than *Nitrososphaera* and consequently leads to their increased relative abundance. Furthermore, numerous studies have shown that types of N sources and the application amount significantly affect the species composition of N-transforming microbial communities (Ma et al., 2018; Xiao et al., 2021). In this study, the relative abundance of the dominant genus within the community of ammonia-oxidizing bacteria was significantly (*p* < 0.05) affected by the amount of N applied, aligning with the findings from Wang’s investigation (Wang et al., 2019), where a higher sensitivity toward N input was observed among ammonia-oxidizing bacteria. Moreover, a high amount of N application resulted in a significant decrease in the relative abundance of the dominant genus of denitrifying bacteria (*p* < 0.05, Figure 2), as it effectively reduced potential N_2_O emissions (Deng et al., 2022). In contrast, the abundance of the dominant genera of all four N-transforming microorganisms was significantly affected by the types of N source (*p* < 0.05), indicating their distinct adaptations to different forms of N.

The addition of N significantly affected the species diversity of soil N-transforming microbial communities in the grasslands, while the different types of grasslands were affected differently by N addition. There was an interaction between the types of N source and the amount of N application on the diversity of soil N conversion microbial communities in the grasslands. The diversity of ammonia-oxidizing archaeal communities in temperate steppe soils was significantly affected by N addition (*p* < 0.05), but neither the types of N source nor the amount of N application had a significant individual effect on them. Soil ammonia-oxidizing bacterial communities of warm-temperate shrub-like soils were significantly (*p* < 0.05) affected by the types of N source, but not significantly (*p* > 0.05, Figure 3) influenced by the interaction of the types of N source and the amount of N application. Competition reduces the species diversity of microbial communities (Smith et al., 2018). In this study, the co-occurrence network analysis revealed a low assortativity of ammonia-oxidizing archaea and ammonia-oxidizing bacterial communities in warm-temperate shrub-like soils (Table 2), indicating internal competition within these communities. This finding elucidates the higher diversity of ammonia-oxidizing archaea and ammonia-oxidizing bacterial communities in the temperate steppe compared with the warm-temperate shrub (Figure 3). In addition, a high amount of ammonium source N application significantly enhanced the diversity of soil ammonia-oxidizing archaea species, whereas a low amount of ammonium source N application led to a significant decrease in both abundance and evenness of soil ammonia-oxidizing bacteria in the temperate steppe. Moreover, a low amount of organic source N application resulted in a reduction in the number of soil denitrifying bacterial species in the warm-temperate shrub (*p* < 0.05). These findings suggest that targeted control over the diversity of N-transforming microbial communities can be achieved by adjusting the amount and type of N applied (Fangliang et al., 2013). Furthermore, the diversity of all four N-transforming microbial communities was significantly affected by the types of N source, indicating that different N-transforming microbial communities exhibited distinct adaptations to various forms of N.

The addition of N significantly influenced the community structure of soil N-transforming microorganisms in the grasslands, whereas different types of grasslands exhibited varying responses to N addition. In this study, soil N-fixing bacterial communities dominated with the most complex, stable, and connected symbiotic network (Table 2). This implies that nitrogen addition may result in a long-term concentration of grassland soil nitrogen cycle through nitrogen fixation processes. Almost all the different soil N-transforming microorganisms in the different grassland types were significantly affected by ammonium type of N addition (*p* < 0.05, Figure 4), suggesting that the ammonium type of N source is the most influential factor driving changes in the structure of the N-transforming microbial community. This could be attributed to its strong influence on soil ammonification rates (Figure 1). Previous studies have demonstrated that high soil ammonification rates can enhance the efficiency of ammonium N utilization (Sun et al., 2020). Different forms of nitrogen exert varying effects on the adaptation of microbial communities involved in nitrogen transformation. In the warm-temperate shrub, soil ammonium-oxidizing archaeal communities were significantly (*p* < 0.05) affected by organic and nitrate types of N application, probably due to their susceptibility to environmental factors (Figure 5). Studies have shown that the amount of N applied is the primary determinant of the soil N-fixing bacterial community structure (Coelho et al., 2008). Our findings indicate that low levels of N addition had a significant impact on the structure of soil N-fixing bacterial communities in the warm-temperate shrub (*p* < 0.05), but no significant effect was observed in the temperate steppe by the amount of N application (*p* > 0.05, Figure 4). The study sites that exhibited a significant correlation with the amount of N application predominantly comprised agricultural lands or soils characterized by high soil nutrient contents. In contrast, soils in the temperate grassland category displayed lower nutrient levels and greater degradation (Supplementary Figure S2), potentially attributable to disparities in the soil-based nutrient conditions. In addition, organic, ammonium, and nitrate types of N and low, medium, and high amounts of N application significantly affected the structure of soil denitrifying bacterial communities in different grassland types (*p* < 0.05). This suggests that denitrifying bacterial communities were primarily impacted by the addition of nitrogen, which is consistent with the finding of Chen et al.’s study, which highlights their heightened responsiveness to environmental disturbances (Chen et al., 2010).

The abundance of microbial communities is affected by external factors and decreases with the decreasing particle size (Chiu et al., 2006). In this study, the grain size of temperate steppe soils was much smaller than that of the warm-temperate shrub (Supplementary Figure S2), which accounts for the lesser impact of environmental factors on the composition of the soil N-fixing bacterial communities in temperate steppe soils (Figure 5). However, the higher rate of soil N conversion in the temperate steppe suggests a faster soil N cycling in the warm-temperate shrub, where nitrification and denitrification processes play a greater role compared with the temperate steppe. This may explain why environmental factors only affect the composition of soil ammonia-oxidizing archaea, ammonia-oxidizing bacteria, and denitrifying bacterial communities in warm-temperate shrubs. Supplementary Table S3 demonstrates that warm-temperate shrub soils exhibit higher stability, indicating their enhanced buffering capacity in protecting microbial cells against environmental fluctuations (Neumann et al., 2013). Furthermore, soil porosity serves as a protective mechanism for preserving the microbial community diversity amidst changing conditions (Xiao et al., 2021). Previous research has revealed an increased occurrence of isolated hydrofilms in soils with larger pores and that soil porosity was greater in sandy soils than in loamy soils (Günal et al., 2018), which may restrict the hydrofilm-mediated protection of the microbial community diversity in temperate grassland-like soils (Chau et al., 2011). This observation also elucidates why the influence of environmental factors on diversity of N-transforming microbial communities is comparatively weaker in warm-temperate shrub-like soils (Figure 5). In addition, the differential response of various N-transforming microorganisms to environmental factors may be attributed to variations in the diversity of soil microorganisms and their distinct reactions to soil minerals. Previous studies have demonstrated that microbial communities with a higher diversity exhibit greater resistance to external influences (Xun et al., 2021), while bacterial, archaeal, and fungal community diversities are differentially affected by mineral composition (Hemkemeyer et al., 2014). The addition of N significantly impacted soil pH, leading to a decrease in pH with higher amounts of N application. It has been reported that an increase in soil pH can create unfavorable conditions for AOA but favorable conditions for AOB (He et al., 2012; Harter et al., 2014). However, the changes in pH resulting from N addition did not significantly affect soil ammonia-oxidizing archaea or ammonia-oxidizing bacterial community diversity in this study, suggesting that alterations in pH due to N deposition do not exert negative effects on the soil microbial community diversity. The application of N in this study was estimated by Dannan Zheng based on a 2010 study of N deposition in China (Zheng et al., 2014) and data from the National Ecological Data Center.^3^ The predicted approximate N deposition for 2020 was set as a medium amount of N, with a low amount of N slightly lower than the 2010 N deposition and a high amount of N slightly higher than the 2030 predicted N deposition. The results of the study showed that none of the changes in environmental factors due to N addition significantly impacted the composition and diversity of the grassland soil N-transforming microbial community but significantly increased the community abundance and diversity (*p* < 0.05, Figure 5). This may imply that, although short-term N deposition would alter the structural composition and diversity of soil N-transforming microbial communities to some extent, it would not have significant negative effects over the next decade.

## 5 Conclusion

In this study, we simulated nitrogen deposition through nitrogen addition experiments, employed high-throughput sequencing technology to analyze the impact of nitrogen deposition on the composition, diversity, and structure of soil nitrogen-cycling microbial communities (nitrogen-fixing bacteria, ammonia-oxidizing archaea, ammonia-oxidizing bacteria, and anti-nitrifying bacteria) in different grassland types in northwestern Liaoning Province, and explored the underlying response mechanisms to environmental changes.

The results showed that N addition significantly affected the structural composition and diversity of soil N-transforming microbial communities in grasslands, albeit with varying affecting mechanisms. Moreover, the response of soil N-cycling microbial communities to N addition differed among various grassland types, with the influence of N source types being more pronounced than that of N application amount. The ammonium type of N source is considered to be the most influential driving factor affecting changes in the structure of the N-transforming microbial community, and the amount of low N applied is the primary determinant governing the structure of the soil N-fixing and nitrifying bacterial community. The findings indicate that short-term N deposition would lead to the dominance of the soil N fixation process in grasslands, thereby exerting a positive effect on the grassland ecosystem in northwest Liaoning. Under the background of increasing nitrogen deposition, investigating the characteristics of soil microbial community and its response mechanism to nitrogen addition in different grassland types in northwest Liaoning plays a crucial role in biodiversity conservation, ecosystem balance maintenance, as well as evaluating and predicting the response of diverse grassland types to global change. However, this study has certain limitations due to factors such as the complex environment in northwest Liaoning, differences between pot experiments and the field conditions, and the relatively short duration of the experiment. In future research, expanding the range of N application levels is recommended to determine the threshold values for positive and negative effects on grasslands. Additionally, further investigation into how different types of grasslands respond and adapt to N deposition can provide valuable insights into understanding patterns related to microbial transformations associated with nitrogen.

## Data availability statement

The DNA sequences used in this study have been deposited in the National Center for Biotechnology Information (NCBI) Sequence Read Archive (SRA) database under the accession number PRJNA929669.

## Author contributions

BR: Conceptualization, Funding acquisition, Methodology, Supervision, Validation, Visualization, Writing – original draft, Writing – review & editing. XM: Conceptualization, Data curation, Investigation, Writing – original draft. DL: Conceptualization, Investigation, Methodology, Writing – original draft. LB: Supervision, Validation, Writing – review & editing. JL: Resources, Supervision, Writing – review & editing. JY: Data curation, Methodology, Writing – original draft. MM: Methodology, Software, Writing – original draft. HL: Investigation, Methodology, Writing – original draft.

## Footnotes

^1^ https://docs.qiime2.org/2019.4/tutorials/

^2^ https://github.com/rdpstaff/Framebot

^3^ http://www.nesdc.org.cn/
