# Supplementary material for: Nitrogen-cycling microbial communities respond differently to nitrogen addition under two contrasting grassland soil types
Source: Front Microbiol. 2024 May 30;15:1290248. doi: 10.3389/fmicb.2024.1290248 (PMC11169941; doi:10.3389/fmicb.2024.1290248)
Supplement: Supplementary file 1 [file Presentation_1.pdf]

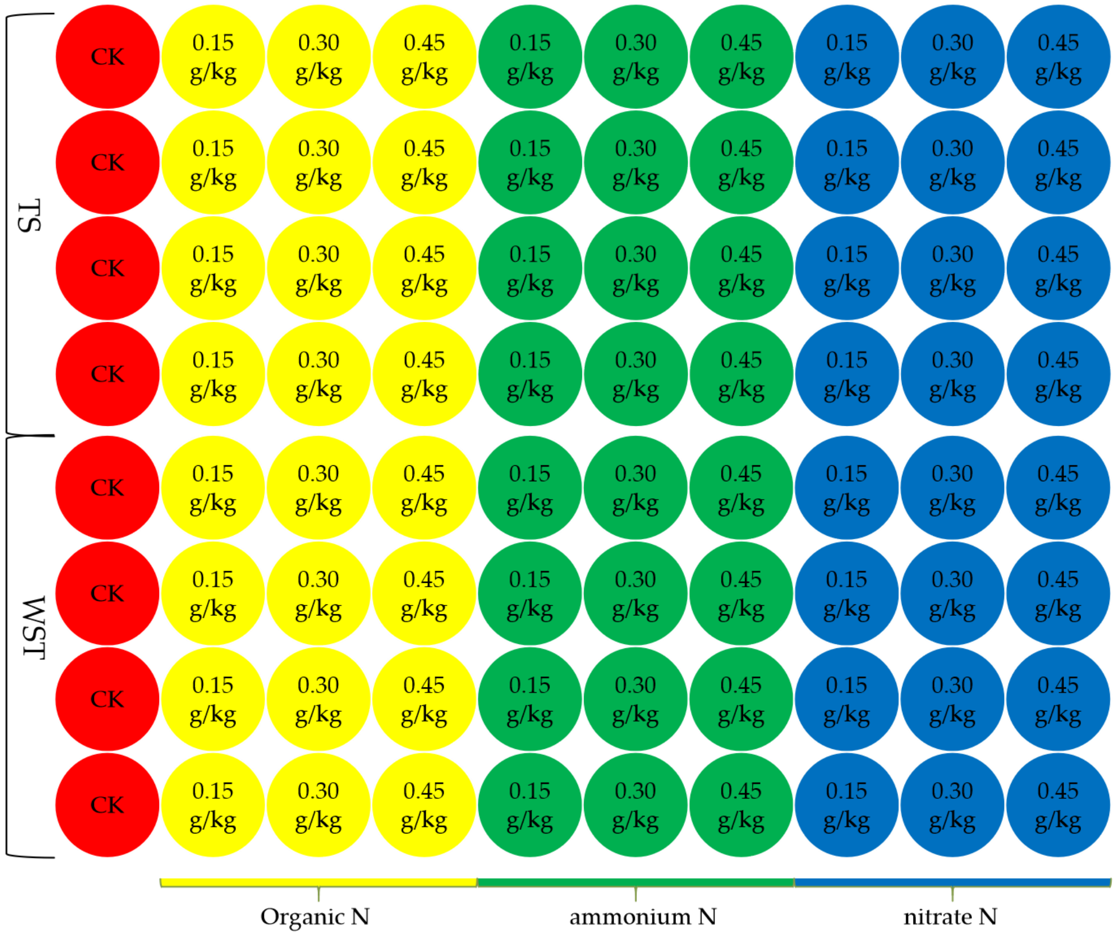

Figure. S1. Schematic diagram of nitrogen addition.

Table S1
Primers used for N-cycling microbial community.

N-cycling microbial community	Primer	Primer sequence(Ke et al., 2013; Yin et al., 2014)
nifH	PolF	5′-TGCGAYCCSAARGCBGACTC-3′
	PolR	5′-ATSGCCATCATYTCRCCGGA-3′
archaeal-amoA	Arch-amoA26F	5′-GACTACATMTTCTAYACWGAYTGGGC-3′
	Arch-amoA417R	5′-GGKGTCATRTATGGWGGYAAYGTTGG-3′
bacterial-amoA	amoA-1F	5′-GGGGTTTCTACTGGTGGT-3′
	amoA-2R	5′-CCCCTCKGSAAAGCCTTCTTC-3′
nirK	FlaCu	5′-ATCATGGTSCTGCCGCG-3′
	R3Cu	5′-GCCTCGATCAGRTTGTGGTT-3

Table S2

Physical and chemical properties of grass soils at 10, 30, 60 and 90 days of incubation.

Grassland type	D10<br>Physical and chemical properties	Nitrogen source	Amount of nitrogen applied<br>0g/kg	0.15g/kg	0.30g/kg	0.45g/kg
		CK	0.16±0.01	0.16±0.01C	0.16±0.01A	0.16±0.01C
	TN (g/kg)	Organic nitrogen	0.16±0.01c	0.29±0.01bB	0.32±0.03abB	0.34±0.04aB
		Ammonium nitrogen	0.16±0.01c	0.37±0.04bA	0.39±0.02bA	0.50±0.11aA
		Nitrate nitrogen	0.16±0.01b	0.19±0.02abC	0.21±0.03aC	0.20±0.01aC
		CK	0.31±0.02	0.31±0.02A	0.31±0.02A	0.31±0.02A
	TP (g/kg)	Organic nitrogen	0.31±0.02a	0.28±0.01abAB	0.29±0abAB	0.26±0.05bB
		Ammonium nitrogen	0.31±0.02a	0.27±0.02abAB	0.26±0.03B	0.26±0.03bB
		Nitrate nitrogen	0.31±0.02a	0.27±0.03bB	0.20±0.03cC	0.21±0.01cB
		CK	1.28±0.14	1.28±0.14aA	1.28±0.14A	1.28±0.14A
	SOC (g/kg)	Organic nitrogen	1.28±0.14a	1.28±0.14aA	1.35±0.23aA	0.98±0.11bB
		Ammonium nitrogen	1.28±0.14a	1.30±0.15aA	0.80±0.03bB	0.75±0.00bC
		Nitrate nitrogen	1.28±0.14a	0.68±0.00bB	0.75±0.08bB	0.75±0.00bC
		CK	5.69±0.05	5.69±0.05A	5.69±0.05B	5.69±0.05B
	pH	Organic nitrogen	5.69±0.05c	4.90±0.17dC	6.79±0.10bA	7.96±0.08aA
		Ammonium nitrogen	5.69±0.05a	5.33±0.20bB	5.61±0.16aB	5.52±0.17abC
		Nitrate nitrogen	5.69±0.05ab	5.79±0.13aA	5.59±0.05bB	5.42±0.04cC
		CK	41.60±7.27	41.60±7.27B	41.60±7.27C	41.60±7.27C
TS	EC (S/m)	Organic nitrogen	41.60±7.27c	160.67±27.80aA	99.38±17.45bB	74.73±9.42bC
		Ammonium nitrogen	41.60±7.27c	147.02±28.69bcA	264.67±6.94bA	694.75±246.87aA
		Nitrate nitrogen	41.60±7.27c	73.05±10.82cB	244.63±33.14bA	335.00±27.07aB
		CK	29.31±5.78	29.31±5.78B	29.31±5.78C	29.31±5.78C
	AN (mg/kg)	Organic nitrogen	29.31±5.78c	33.83±4.37cB	110.69±18.60bB	147.88±14.11aB
		Ammonium nitrogen	29.31±5.78d	111.56±34.04cA	150.5±18.68bA	469.88±9.29aA
		Nitrate nitrogen	29.31±5.78	27.13±4.63B	28.00±4.29C	25.08±1.65C
		CK	3.09±0.72	3.09±0.72C	3.09±0.72C	3.09±0.72C
	NH_4^+-N (mg/kg)	Organic nitrogen	3.09±0.72d	81.03±11.52cB	171.73±24.03bA	221.01±18.69aB
		Ammonium nitrogen	3.09±0.72c	104.51±8.7bA	141.06±25.24bB	305.26±46.69aA
		Nitrate nitrogen	3.09±0.72b	4.70±0.81aC	4.60±0.60aC	4.37±0.64aC
		CK	5.72±1.26	5.72±1.26B	5.72±1.26B	5.72±1.26B
	NO_3^--N (mg/kg)	Organic nitrogen	5.72±1.26a	13.02±2.48bB	21.51±0.36aB	4.16±1.54aB
		Ammonium nitrogen	5.72±1.26ab	6.28±1.10aB	3.96±1.27bB	6.29±1.85aB
		Nitrate nitrogen	5.72±1.26d	69.18±11.09cA	112.77±22.15bA	241.17±21.54aA
		CK	22.65±1.11	22.65±1.11A	22.65±1.11	22.65±1.11A
	SP (mg/kg)	Organic nitrogen	22.65±1.11	22.87±1.72A	21.98±1.25	21.42±1.61AB
		Ammonium nitrogen	22.65±1.11ab	25.26±2.71aA	21.49±2.36b	21.71±1.54bAB

		Nitrate nitrogen	22.65±1.11a	19.47±2.05bB	21.79±1.48ab	19.62±1.02bB
	ON (mg/kg)	CK	0.15±0.01	0.15±0.01C	0.15±0.01B	0.15±0.01
		Organic nitrogen	0.15±0.01ab	0.21±0.02aB	0.15±0.04abB	0.12±0.06b
		Ammonium nitrogen	0.15±0.01	0.26±0.05A	0.25±0.04A	0.19±0.14
		Nitrate nitrogen	0.15±0.01b	0.18±0.02abBC	0.21±0.04aA	0.20±0.01a
WST	TN (g/kg)	CK	1.05±0.01	1.05±0.01B	1.05±0.01B	1.05±0.01D
		Organic nitrogen	1.05±0.01c	1.21±0.06bA	1.33±0.09aA	1.39±0.07aB
		Ammonium nitrogen	1.05±0.01c	1.09±0.07cB	1.28±0.04bA	1.54±0.06aA
		Nitrate nitrogen	1.05±0.01	1.10±0.08B	1.12±0.14B	1.15±0.07C
	TP (g/kg)	CK	0.67±0.03	0.67±0.03B	0.67±0.03B	0.67±0.03B
		Organic nitrogen	0.67±0.03b	0.69±0.05abAB	0.69±0.05abB	0.76±0.05aA
		Ammonium nitrogen	0.67±0.03ab	0.72±0.03abAB	0.66±0.02bB	0.73±0.06aAB
		Nitrate nitrogen	0.67±0.03c	0.74±0.01bA	0.79±0.03aA	0.73±0.02bAB
	SOC (g/kg)	CK	10.01±0.52	10.01±0.52A	10.01±0.52	10.01±0.52A
		Organic nitrogen	10.01±0.52	9.76±0.63AB	9.68±0.72	9.44±0.48A
		Ammonium nitrogen	10.01±0.52a	8.87±0.43bB	9.7±0.73ab	9.91±0.64aA
		Nitrate nitrogen	10.01±0.52a	10.58±0.93aA	10.33±0.57a	7.94±0.08bB
	pH	CK	7.20±0.16	7.20±0.16A	7.20±0.16A	7.20±0.16AB
		Organic nitrogen	7.20±0.16b	7.08±0.11bAB	7.02±0.08bAB	7.81±0.20aA
		Ammonium nitrogen	7.20±0.16a	6.94±0.13bB	6.97±0.14bB	7.02±0.05abBC
		Nitrate nitrogen	7.20±0.16a	7.17±0.17abAB	6.98±0.09bcB	6.94±0.07cC
	EC (S/m)	CK	86.18±18.69	86.18±18.69C	86.18±18.69C	86.18±18.69C
		Organic nitrogen	86.18±18.69c	259.75±26.99bA	380.50±25.48aB	226.60±32.27bBC
		Ammonium nitrogen	86.18±18.69c	267.00±28.01cA	616.50±164.59bA	994.25±368.83aA
		Nitrate nitrogen	86.18±18.69d	181.33±19.97cB	675.00±16.08aA	481.25±72.83bB
	AN (mg/kg)	CK	83.56±8.38	83.56±8.38C	83.56±8.38C	83.56±8.38C
		Organic nitrogen	83.56±8.38c	126.44±25.5cB	203.88±52.35bB	373.63±60.83aB
		Ammonium nitrogen	83.56±8.38d	190.17±14.66cA	426.13±47.57bA	548.92±73.78aA
		Nitrate nitrogen	83.56±8.38b	84.00±13.48bC	98.58±5.41aC	81.81±6.91bC
	NH_4^+-N (mg/kg)	CK	3.15±0.05	3.15±0.05C	3.15±0.05C	3.15±0.05C
		Organic nitrogen	3.15±0.05c	65.14±7.92bB	200.27±44.47aA	205.58±10.87aB
		Ammonium nitrogen	3.15±0.05c	106.24±32.43bA	138.86±26.89bB	448.98±70.57aA
		Nitrate nitrogen	3.15±0.05b	4.35±0.76aC	4.18±0.88abC	4.07±0.62abC
	NO_3^--N (mg/kg)	CK	19.52±2.82	19.52±2.82B	19.52±2.82B	19.52±2.82B
		Organic nitrogen	19.52±2.82	15.44±0.34B	16.77±4.49B	15.54±1.92B
		Ammonium nitrogen	19.52±2.82b	17.29±1.84bB	27.01±2.78aB	30.31±6.20aB
		Nitrate nitrogen	19.52±2.82d	135.9±24.16cA	235.02±42.96bA	349.03±56.13aA
	SP (mg/kg)	CK	3.82±0.63	3.82±0.63	3.82±0.63	3.82±0.63
		Organic nitrogen	3.82±0.63	3.22±0.58	3.18±0.49	2.81±0.79
		Ammonium nitrogen	3.82±0.63	3.22±0.50	3.59±1.13	3.37±0.48

		Nitrate nitrogen	3.82±0.63	3.26±0.51	2.88±0.56	2.96±1.33
		CK	1.05±0.01	1.05±0.01AB	1.05±0.01	1.05±0.01B
	ON	Organic nitrogen	1.05±0.01b	1.15±0.07abA	1.13±0.10ab	1.18±0.07aA
	(mg/kg)	Ammonium nitrogen	1.05±0.01ab	0.99±0.10bB	1.14±0.04a	1.09±0.07abAB
		Nitrate nitrogen	1.05±0.01	1.10±0.08AB	1.11±0.14	1.14±0.07AB

**Grassland type**	**D30 Physical and chemical properties**	**Nitrogen source**	**Amount of nitrogen applied**			
			**0g/kg**	**0.15g/kg**	**0.30g/kg**	**0.45g/kg**
		CK	0.17±0.00	0.17±0.00B	0.17±0.00B	0.17±0.00C
	TN	Organic nitrogen	0.17±0.00a	0.20±0.02bB	0.26±0.03cB	0.29±0.01dB
	(g/kg)	Ammonium nitrogen	0.17±0.00c	0.27±0.05bcA	0.35±0.11bA	0.51±0.11aA
		Nitrate nitrogen	0.17±0.00	0.17±0.02B	0.18±0.03B	0.19±0.01C
		CK	0.26±0.01	0.26±0.01	0.26±0.01C	0.26±0.01B
	TP	Organic nitrogen	0.26±0.01	0.29±0.03	0.29±0.02BC	0.30±0.05AB
	(g/kg)	Ammonium nitrogen	0.26±0.01b	0.30±0.03a	0.33±0.01aA	0.26±0.03bB
		Nitrate nitrogen	0.26±0.01c	0.28±0.02bc	0.30±0.03abAB	0.32±0.03aA
		CK	1.75±0.17	1.75±0.17A	1.75±0.17A	1.75±0.17A
	SOC	Organic nitrogen	1.75±0.17	1.62±0.31AB	1.41±0.15BC	1.44±0.24A
	(g/kg)	Ammonium nitrogen	1.75±0.17a	1.35±0.20bBC	1.48±0.22abB	1.46±0.19abA
		Nitrate nitrogen	1.75±0.17a	1.21±0.06bB	1.20±0.04bC	0.88±0.35cB
		CK	6.24±0.11	6.24±0.11A	6.24±0.11B	6.24±0.11B
		Organic nitrogen	6.24±0.11c	5.56±0.23dB	6.67±0.18bA	7.46±0.18aA
	pH	Ammonium nitrogen	6.24±0.11a	5.51±0.19cB	5.60±0.10cC	5.94±0.23bC
TS		Nitrate nitrogen	6.24±0.11a	6.36±0.22aA	6.19±0.17aB	5.88±0.21bC
		CK	43.73±6.36	43.73±6.36C	43.73±6.36B	43.73±6.36C
	EC	Organic nitrogen	43.73±6.36b	83.08±26.46abBC	107.70±50.75aB	73.00±10.55abC
	(S/m)	Ammonium nitrogen	43.73±6.36b	154.15±35.91bA	308.55±107.09aA	378.93±100.97aA
		Nitrate nitrogen	43.73±6.36c	91.75±24.18cB	152.90±65.98bB	295.00±8.52aB
		CK	21.44±3.88	21.44±3.88B	21.44±3.88C	21.44±3.88C
	AN	Organic nitrogen	21.44±3.88c	31.06±9.52cB	77.88±12.82bB	112.44±21.26aB
	(mg/kg)	Ammonium nitrogen	21.44±3.88c	108.94±14.74bA	261.33±26.55aA	303.63±85.38aA
		Nitrate nitrogen	21.44±3.88	23.63±8.39B	20.13±3.03C	17.50±1.43C
		CK	0.75±0.35	0.75±0.35B	0.75±0.35C	0.75±0.35C
	NH_4^+-N	Organic nitrogen	0.75±0.35c	19.48±7.36cB	67.72±15.52bB	124.82±17.96aB
	(mg/kg)	Ammonium nitrogen	0.75±0.35c	133.31±59.04bA	203.89±18.62aA	257.87±64.79aA
		Nitrate nitrogen	0.75±0.35c	2.59±0.62bB	2.32±1.03bC	4.36±0.67aC
		CK	17.12±1.49	17.12±1.49BC	17.12±1.49BC	17.12±1.49B
	NO_3^--N	Organic nitrogen	17.12±1.49c	25.47±6.11bcB	32.16±8.50bB	42.07±4.96aB
	(mg/kg)	Ammonium nitrogen	17.12±1.49a	12.80±0.67bC	8.20±2.01cC	8.91±1.83cB

		Nitrate nitrogen	17.12±1.49c	47.88±10.36cA	102.9±21.41bA	222.48±47.42aA
	SP (mg/kg)	CK	23.43±2.13	23.43±2.13	23.43±2.13	23.43±2.13
		Organic nitrogen	23.43±2.13	22.5±1.26	23.4±2.48	23.36±2.29
		Ammonium nitrogen	23.43±2.13	23.17±2.17	19.36±4.55	21.56±1.98
		Nitrate nitrogen	23.43±2.13	23.55±1.65	23.1±1.21	22.65±3.38
	ON (mg/kg)	CK	0.16±0.00	0.16±0.00	0.16±0.00	0.16±0.00
		Organic nitrogen	0.16±0.00	0.18±0.02	0.19±0.04	0.16±0.01
		Ammonium nitrogen	0.16±0.00	0.18±0.04	0.15±0.13	0.25±0.15
		Nitrate nitrogen	0.16±0.00	0.17±0.02	0.18±0.03	0.18±0.01
WST	TN (g/kg)	CK	1.02±0.02	1.02±0.02B	1.02±0.02B	1.02±0.02C
		Organic nitrogen	1.02±0.02c	1.21±0.05bA	1.51±0.18aA	1.61±0.05aB
		Ammonium nitrogen	1.02±0.02d	1.19±0.05cA	1.46±0.1bA	1.92±0.15aA
		Nitrate nitrogen	1.02±0.02	1.03±0.06	1.20±0.23B	1.06±0.09C
	TP (g/kg)	CK	0.71±0.09	0.71±0.09B	0.71±0.09	0.71±0.09
		Organic nitrogen	0.71±0.09	0.75±0.04B	0.71±0.10	0.74±0.02
		Ammonium nitrogen	0.71±0.09	0.69±0.03B	0.73±0.12	0.75±0.09
		Nitrate nitrogen	0.71±0.09c	0.85±0.02abA	0.87±0.08a	0.76±0.03bc
	SOC (g/kg)	CK	9.78±0.82	9.78±0.82A	9.78±0.82A	9.78±0.82A
		Organic nitrogen	9.78±0.82	9.92±0.13A	9.7±0.93A	9.40±0.64A
		Ammonium nitrogen	9.78±0.82a	8.58±0.32bB	8.29±0.61bB	8.55±0.37bB
		Nitrate nitrogen	9.78±0.82a	8.50±0.71bB	10.51±0.76aA	7.73±0.07bB
	pH	CK	7.33±0.17	7.33±0.17	7.33±0.17A	7.33±0.17B
		Organic nitrogen	7.33±0.17ab	7.13±0.09bc	7.10±0.05cB	7.48±0.17aA
		Ammonium nitrogen	7.33±0.17a	7.19±0.11a	6.85±0.12bC	6.73±0.15bA
		Nitrate nitrogen	7.33±0.17a	7.17±0.19ab	7.06±0.05bB	7.00±0.06bC
	EC (S/m)	CK	86.17±18.94	86.17±18.94B	86.17±18.94C	86.17±18.94B
		Organic nitrogen	86.17±18.94c	224.43±61.16abA	297.25±87.86aB	172.88±20.09cB
		Ammonium nitrogen	86.17±18.94c	205.48±47.06cA	460.25±128.62bA	1055.25±264.18aA
		Nitrate nitrogen	86.17±18.94d	268.50±97.58cA	451.00±31.29bA	876.75±211.39aA
	AN (mg/kg)	CK	92.31±2.99	92.31±2.99C	92.31±2.99C	92.31±2.99C
		Organic nitrogen	92.31±2.99c	119.00±2.02cB	160.56±8.62bB	227.5±52.93aB
		Ammonium nitrogen	92.31±2.99c	144.81±14.67cA	225.31±67.09bA	381.50±39.31aA
		Nitrate nitrogen	92.31±2.99a	93.92±2.97aC	81.08±15.28abC	75.25±5.72bC
	NH_4^+-N (mg/kg)	CK	0.98±0.27	0.98±0.27B	0.98±0.27C	0.98±0.27C
		Organic nitrogen	0.98±0.27c	3.96±0.84cB	30.07±6.88bB	65.22±16.83aB
		Ammonium nitrogen	0.98±0.27c	45.93±13.66cA	173.18±32.77bA	271.66±66.33aA
		Nitrate nitrogen	0.98±0.27	1.63±0.95B	1.15±0.90C	1.27±0.08C
	NO_3^--N (mg/kg)	CK	22.89±2.54	22.89±2.54B	22.89±2.54B	22.89±2.54C
		Organic nitrogen	22.89±2.54c	133±33.24bA	194.61±40.33aA	127.87±33.88bB
		Ammonium nitrogen	22.89±2.54c	42.77±13.06bcB	53.66±21.39abB	66.28±14.28aC

		Nitrate nitrogen	22.89±2.54c	144.5±35.79bA	187.88±38.87bA	353.07±61.17aA
		CK	2.73±0.94	2.73±0.94	2.73±0.94	2.73±0.94
	SP (mg/kg)	Organic nitrogen	2.73±0.94	2.77±0.50	2.4±0.63	3.59±1.05
		Ammonium nitrogen	2.73±0.94	1.95±0.63	2.44±1.02	3.15±0.70
		Nitrate nitrogen	2.73±0.94	2.47±0.58	3.15±0.47	3.03±0.49
		CK	1.02±0.02	1.02±0.02B	1.02±0.02C	1.02±0.02C
	ON (mg/kg)	Organic nitrogen	1.02±0.02c	1.21±0.05bA	1.48±0.18aA	1.55±0.05aB
		Ammonium nitrogen	1.02±0.02d	1.15±0.04cA	1.29±0.08bAB	1.65±0.09aA
		Nitrate nitrogen	1.02±0.02	1.02±0.06B	1.20±0.23BC	1.06±0.09C

Grassland type	D60 Physical and chemical properties	Nitrogen source	Amount of nitrogen applied 0g/kg	0.15g/kg	0.30g/kg	0.45g/kg
TS	TN (g/kg)	CK	0.15±0.01	0.15±0.01C	0.15±0.01B	0.15±0.01C
		Organic nitrogen	0.15±0.01b	0.20±0.02aAB	0.20±0.03aB	0.23±0.02aAB
		Ammonium nitrogen	0.15±0.01c	0.26±0.05bA	0.31±0.10abA	0.39±0.07aA
		Nitrate nitrogen	0.15±0.01	0.17±0.01BC	0.18±0.03B	0.18±0.04BC
	TP (g/kg)	CK	0.10±0.02	0.10±0.02B	0.10±0.02B	0.10±0.02B
		Organic nitrogen	0.10±0.02ab	0.05±0.03bB	0.14±0.06abB	0.13±0.04aB
		Ammonium nitrogen	0.10±0.02b	0.18±0.05aA	0.14±0.06abB	0.20±0.04aA
		Nitrate nitrogen	0.10±0.02b	0.22±0.04aA	0.24±0.02aA	0.24±0.04aA
	SOC (g/kg)	CK	2.13±0.22	2.13±0.22A	2.13±0.22A	2.13±0.22A
		Organic nitrogen	2.13±0.22a	1.72±0.13bB	1.57±0.27bAB	1.92±0.28abB
		Ammonium nitrogen	2.13±0.22	1.43±0.08C	1.27±0.97B	1.32±1.11BC
		Nitrate nitrogen	2.13±0.22a	0.57±0.22bD	0.74±0.15bC	0.56±0.18bC
	pH	CK	6.11±0.18	6.11±0.18AB	6.11±0.18A	6.11±0.18AB
		Organic nitrogen	6.11±0.18a	5.73±0.22bBC	5.57±0.19bB	5.64±0.23bBC
		Ammonium nitrogen	6.11±0.18a	5.31±0.49bC	5.02±0.38bC	5.32±0.27bC
		Nitrate nitrogen	6.11±0.18	6.31±0.15A	6.04±0.28A	6.40±0.62A
	EC (S/m)	CK	45.20±11.93	45.20±11.93B	45.20±11.93C	45.20±11.93C
		Organic nitrogen	45.20±11.93b	71.90±34.78abB	91.40±23.79aBC	79.60±15.69abBC
		Ammonium nitrogen	45.20±11.93b	156.90±41.03bA	314.30±58.02aA	403.10±134.36aA
		Nitrate nitrogen	45.20±11.93b	87.00±15.43bB	133.70±45.31aB	176.93±34.35aC
	AN (mg/kg)	CK	21.44±2.99	21.44±2.99B	21.44±2.99B	21.44±2.99B
		Organic nitrogen	21.44±2.99b	24.5±2.47bB	27.13±0.71bB	34.13±5.98aB
		Ammonium nitrogen	21.44±2.99b	102.81±15.01bA	228.08±58.61aA	281.75±88.09aA
		Nitrate nitrogen	21.44±2.99	22.75±4.29B	27.56±9.08B	23.63±7.89B
	NH_4^+-N (mg/kg)	CK	1.89±0.35	1.89±0.35B	1.89±0.35B	1.89±0.35B
		Organic nitrogen	1.89±0.35c	2.07±0.71cB	18.43±1.52bB	46.22±2.37aB
		Ammonium nitrogen	1.89±0.35b	62.29±24.12bA	180.77±22.70aA	243.18±108.50aA

		Nitrate nitrogen	1.89±0.35	2.02±0.86B	3.07±1.07B	2.86±0.36B
	NO_3^--N (mg/kg)	CK	2.88±0.07	2.88±0.07C	2.88±0.07C	2.88±0.07C
		Organic nitrogen	2.88±0.07c	8.63±1.03cB	22.55±0.88bB	42.77±8.49aB
		Ammonium nitrogen	2.88±0.07b	18.11±5.43aA	19.2±6.79aB	20.43±2.70aC
		Nitrate nitrogen	2.88±0.07c	19.5±3.93cA	93.38±13.66bA	121.22±27.4aA
	SP (mg/kg)	CK	20.96±0.45	20.96±0.45	20.96±0.45A	20.96±0.45
		Organic nitrogen	20.96±0.45	17.98±2.75	18.02±1.14AB	20.86±6.03
		Ammonium nitrogen	20.96±0.45a	17.42±2.01b	20.07±1.65abA	19.62±2.51ab
		Nitrate nitrogen	20.96±0.45	21.94±9.00	16.58±3.15B	15.92±1.72
	ON (mg/kg)	CK	0.15±0.01	0.15±0.01B	0.15±0.01	0.15±0.01
		Organic nitrogen	0.15±0.01b	0.20±0.02aAB	0.18±0.03ab	0.19±0.02a
		Ammonium nitrogen	0.15±0.01	0.20±0.04A	0.13±0.10	0.14±0.06
		Nitrate nitrogen	0.15±0.01	0.17±0.01AB	0.18±0.03	0.17±0.04
WST	TN (g/kg)	CK	1.00±0.04	1.00±0.04	1.00±0.04B	1.00±0.04C
		Organic nitrogen	1.00±0.04b	1.07±0.08ab	1.17±0.13aA	1.20±0.14aB
		Ammonium nitrogen	1.00±0.04c	1.04±0.09c	1.25±0.10bA	1.46±0.13aA
		Nitrate nitrogen	1.00±0.04b	1.02±0.08b	1.15±0.06aA	1.08±0.00abBC
	TP (g/kg)	CK	0.82±0.05	0.82±0.05A	0.82±0.05A	0.82±0.05A
		Organic nitrogen	0.82±0.05	0.80±0.05A	0.78±0.02A	0.78±0.09AB
		Ammonium nitrogen	0.82±0.05a	0.63±0.03bB	0.66±0.09bB	0.70±0.07bB
		Nitrate nitrogen	0.82±0.05a	0.69±0.05bB	0.79±0.06aA	0.81±0.05aAB
	SOC (g/kg)	CK	9.86±0.70	9.86±0.70A	9.86±0.70AB	9.86±0.70A
		Organic nitrogen	9.86±0.70a	8.63±0.21bB	8.56±0.41bC	8.44±0.24bB
		Ammonium nitrogen	9.86±0.70a	7.48±0.26bC	9.29±0.8aBC	9.80±0.23aA
		Nitrate nitrogen	9.86±0.70b	10.46±0.87aA	10.57±0.52aA	8.92±0.52abB
	pH	CK	6.92±0.04	6.92±0.04A	6.92±0.04A	6.92±0.04A
		Organic nitrogen	6.92±0.04a	6.70±0.14bA	6.42±0.12cB	6.28±0.19cC
		Ammonium nitrogen	6.92±0.04a	6.03±0.22bB	6.00±0.14bC	5.65±0.16cB
		Nitrate nitrogen	6.92±0.04a	6.74±0.12abA	6.78±0.10abA	6.71±0.18bA
	EC (S/m)	CK	184.45±64.27	184.45±64.27BC	184.45±64.27C	184.45±64.27C
		Organic nitrogen	184.45±64.27b	151.67±14.22bC	330.38±133.38aB	251±40.01abC
		Ammonium nitrogen	184.45±64.27c	382.00±50.04bcA	599.00±104.62bA	1000.75±263.07aA
		Nitrate nitrogen	184.45±64.27c	249.00±87.51cB	569.33±17.33bA	732.50±150.59aB
	AN (mg/kg)	CK	95.81±6.61	95.81±6.61BC	95.81±6.61C	95.81±6.61C
		Organic nitrogen	95.81±6.61b	129.06±36.75aAB	142.63±16.75aB	139.13±11.56aB
		Ammonium nitrogen	95.81±6.61c	132.56±21.59cA	236.69±43.28bA	319.67±40.82aA
		Nitrate nitrogen	95.81±6.61	90.56±5.96C	94.94±17.05C	81.67±1.65C
	NH_4^+-N (mg/kg)	CK	1.30±0.51	1.30±0.51B	1.30±0.51B	1.30±0.51B
		Organic nitrogen	1.30±0.51c	2.83±0.41cB	5.49±1.19bB	13.24±2.34aB
		Ammonium nitrogen	1.30±0.51c	18.54±2.30cA	93.68±30.98bA	279.3±59.17aA

		Nitrate nitrogen	1.30±0.51	1.54±0.46B	1.45±0.42B	1.66±0.58B
		CK	28.60±5.52	28.60±5.52B	28.60±5.52C	28.60±5.52C
	NO_3^--N	Organic nitrogen	28.60±5.52c	39.85±7.36cB	166.15±51.27aA	123.71±16.88bB
	(mg/kg)	Ammonium nitrogen	28.60±5.52b	113.11±71.31aA	95.46±34.87aB	153.52±32.89aB
		Nitrate nitrogen	28.60±5.52b	82.42±46.80bAB	207.91±53.01aA	227.56±45.92aA
		CK	3.15±0.69	3.15±0.69	3.15±0.69B	3.15±0.69
	SP	Organic nitrogen	3.15±0.69	3.22±0.69	3.48±0.56A	3.71±0.61
	(mg/kg)	Ammonium nitrogen	3.15±0.69	2.62±0.83	3.11±0.66B	3.71±0.61
		Nitrate nitrogen	3.15±0.69bc	3.03±0.47c	4.23±0.47aAB	3.97±0.5ab
		CK	0.99±0.04	0.99±0.04	0.99±0.04B	0.99±0.04B
	ON	Organic nitrogen	0.99±0.04b	1.07±0.08ab	1.16±0.13abA	1.19±0.14aA
	(mg/kg)	Ammonium nitrogen	0.99±0.04b	1.02±0.10b	1.15±0.10aA	1.18±0.08aA
		Nitrate nitrogen	0.99±0.04b	1.02±0.08b	1.15±0.06aA	1.08±0.00abAB

**Grassland type**	**D90 Physical and chemical properties**	**Nitrogen source**	**Amount of nitrogen applied** **0g/kg**	**0.15g/kg**	**0.30g/kg**	**0.45g/kg**
TS	TN (g/kg)	CK	0.15±0.01	0.15±0.01C	0.15±0.01C	0.15±0.01B
		Organic nitrogen	0.15±0.01b	0.16±0.01bC	0.18±0.01abBC	0.20±0.03aB
		Ammonium nitrogen	0.15±0.01b	0.24±0.03bA	0.33±0.07aA	0.39±0.08aA
		Nitrate nitrogen	0.15±0.01b	0.19±0.02aB	0.23±0.03aAB	0.20±0.02aB
	TP (g/kg)	CK	0.27±0.08	0.27±0.08	0.27±0.08AB	0.27±0.08
		Organic nitrogen	0.27±0.08	0.31±0.02	0.32±0.03A	0.22±0.11
		Ammonium nitrogen	0.27±0.08	0.23±0.11	0.21±0.12AB	0.21±0.10
		Nitrate nitrogen	0.27±0.08a	0.26±0.02ab	0.17±0.07bB	0.21±0.05ab
	SOC (g/kg)	CK	0.83±0.22	0.83±0.22B	0.83±0.22C	0.83±0.22B
		Organic nitrogen	0.83±0.22c	1.08±0.09abB	0.93±0.12bcB	1.21±0.08aA
		Ammonium nitrogen	0.83±0.22b	1.61±0.22aA	1.49±0.52aA	1.33±0.39abA
		Nitrate nitrogen	0.83±0.22b	1.14±0.42abB	1.28±0.05aAB	1.22±0.00aA
	pH	CK	6.06±0.10	6.06±0.10B	6.06±0.10B	6.06±0.10A
		Organic nitrogen	6.06±0.10a	5.23±0.05cC	5.49±0.27bcC	5.54±0.19bB
		Ammonium nitrogen	6.06±0.10a	4.90±0.15cD	5.03±0.10bcD	5.21±0.17bB
		Nitrate nitrogen	6.06±0.10b	6.60±0.22aA	6.47±0.15abA	6.30±0.50abA
	EC (S/m)	CK	47.33±6.64	47.33±6.64B	47.33±6.64B	47.33±6.64C
		Organic nitrogen	47.33±6.64b	63.93±8.29abB	88.60±14.18aB	86.20±37.54aBC
		Ammonium nitrogen	47.33±6.64d	160.10±18.10cA	323.03±98.55bA	436.33±46.58aA
		Nitrate nitrogen	47.33±6.64b	83.93±49.54bB	112.03±39.27aB	137.48±29.60aB
	AN (mg/kg)	CK	27.42±3.60	27.42±3.60B	27.42±3.60B	27.42±3.60B
		Organic nitrogen	27.42±3.60b	32.38±3.03abB	33.83±4.59abB	41.56±10.34aB

		Ammonium nitrogen	27.42±3.60d	73.5±17.38cA	156.92±36.75bA	250.25±34.91aA
		Nitrate nitrogen	27.42±3.60	28.00±3.78B	31.06±9.63B	32.08±0.82B
		CK	0.87±0.11	0.87±0.11B	0.87±0.11B	0.87±0.11C
	NH_4^+-N	Organic nitrogen	0.87±0.11b	1.36±0.32bB	4.63±2.48bB	17.00±5.93aB
	(mg/kg)	Ammonium nitrogen	0.87±0.11d	49.13±18.23cA	126.63±20.01bA	227.98±8.74aA
		Nitrate nitrogen	0.87±0.11b	1.68±0.50aB	2.05±0.53aB	1.49±0.01aC
		CK	2.58±0.50	2.58±0.50B	2.58±0.50D	2.58±0.50C
	NO_3^--N	Organic nitrogen	2.58±0.50c	6.57±2.13cB	33.01±12.57bB	51.99±0.85aA
	(mg/kg)	Ammonium nitrogen	2.58±0.50b	24.47±10.49aA	18.46±8.12aC	28.86±3.50aB
		Nitrate nitrogen	2.58±0.50c	29.10±10.05bA	50.39±7.15aA	53.97±7.66aA
		CK	14.73±0.84	14.73±0.84B	14.73±0.84B	14.73±0.84B
	SP	Organic nitrogen	14.73±0.84	18.09±3.43A	16.06±1.62AB	18.02±1.86A
	(mg/kg)	Ammonium nitrogen	14.73±0.84b	18.69±1.60aA	18.13±0.86aA	17.47±1.21aA
		Nitrate nitrogen	14.73±0.84b	16.78±0.94abAB	15.34±2.77bB	18.00±0.57aA
		CK	0.15±0.01	0.15±0.01B	0.15±0.01	0.15±0.01
	ON	Organic nitrogen	0.15±0.01b	0.16±0.01bB	0.17±0.01ab	0.19±0.02a
	(mg/kg)	Ammonium nitrogen	0.15±0.01	0.19±0.01A	0.21±0.08	0.16±0.08
		Nitrate nitrogen	0.15±0.01b	0.19±0.02aA	0.22±0.03a	0.20±0.02a
		CK	1.00±0.07	1.00±0.07	1.00±0.07B	1.00±0.07B
	TN	Organic nitrogen	1.00±0.07c	1.10±0.01b	1.23±0.05aA	1.22±0.09aA
	(g/kg)	Ammonium nitrogen	1.00±0.07c	1.04±0.08bc	1.17±0.13abAB	1.28±0.06aA
		Nitrate nitrogen	1.00±0.07	1.02±0.07	1.11±0.19BC	1.04±0.05B
		CK	0.78±0.09	0.78±0.09	0.78±0.09	0.78±0.09A
	TP	Organic nitrogen	0.78±0.09	0.71±0.12	0.75±0.06	0.80±0.06A
	(g/kg)	Ammonium nitrogen	0.78±0.09	0.68±0.16	0.68±0.1	0.69±0.03AB
		Nitrate nitrogen	0.78±0.09a	0.73±0.06a	0.78±0.01a	0.59±0.12bB
		CK	10.96±1.09	10.96±1.09A	10.96±1.09A	10.96±1.09A
	SOC	Organic nitrogen	10.96±1.09	9.96±0.27AB	10.21±0.28A	10.04±0.44AB
	(g/kg)	Ammonium nitrogen	10.96±1.09a	9.15±0.28bB	8.71±1.12bB	9.15±0.50bBC
WST		Nitrate nitrogen	10.96±1.09a	9.27±0.80bcB	9.75±0.05bAB	8.41±0.06cC
		CK	6.92±0.13	6.92±0.13A	6.92±0.13A	6.92±0.13A
		Organic nitrogen	6.92±0.13a	6.80±0.16abA	6.60±0.17abB	6.50±0.33bB
	pH	Ammonium nitrogen	6.92±0.13a	6.30±0.36bB	5.59±0.20cC	5.37±0.21cC
		Nitrate nitrogen	6.92±0.13	6.81±0.20A	6.93±0.12A	6.85±0.08A
		CK	178.03±12.25	178.03±12.25	178.03±12.25B	178.03±12.25C
	EC	Organic nitrogen	178.03±12.25b	262.18±112.67ab	472.75±228.47aA	392.00±119.09abB
	(S/m)	Ammonium nitrogen	178.03±12.25b	253.05±74.37b	562.25±132.58aA	673.50±117.5aA
		Nitrate nitrogen	178.03±12.25c	155.80±18.78c	443.50±78.57bA	621.75±134.09aA
	AN	CK	64.75±15.32	64.75±15.32C	64.75±15.32C	64.75±15.32C
	(mg/kg)	Organic nitrogen	64.75±15.32c	118.13±13.13bA	118.13±14.75bB	146.13±20.63aB

	Ammonium nitrogen	64.75±15.32d	111.13±11.83cAB	182.58±21.40bA	256.81±36.83aA
	Nitrate nitrogen	64.75±15.32c	94.06±5.23abB	109.81±22.52aB	80.94±1.68bcC
	CK	1.32±0.56	1.32±0.56B	1.32±0.56B	1.32±0.56B
NH_4^+-N	Organic nitrogen	1.32±0.56d	2.80±1.00cA	4.77±0.76bB	6.60±0.93aB
(mg/kg)	Ammonium nitrogen	1.32±0.56b	3.06±0.52bA	45.64±12.37bA	120.92±59.33aA
	Nitrate nitrogen	1.32±0.56	2.28±0.17AB	4.36±5.24B	2.09±0.82B
	CK	22.01±4.31	22.01±4.31D	22.01±4.31C	22.01±4.31D
NO_3^--N	Organic nitrogen	22.01±4.31b	240.10±26.97aA	302.42±67.02aA	262.01±60.87aB
(mg/kg)	Ammonium nitrogen	22.01±4.31c	54.15±13.21bC	110.40±14.23aB	118.03±19.23aC
	Nitrate nitrogen	22.01±4.31d	98.63±10.22cB	246.28±65.69bA	343.70±66.67aA
	CK	2.81±0.29	2.81±0.29	2.81±0.29	2.81±0.29B
SP	Organic nitrogen	2.81±0.29b	2.92±0.48b	3.33±0.71ab	4.13±0.6aA
(mg/kg)	Ammonium nitrogen	2.81±0.29	3.43±0.64	3.03±0.12	3.26±0.64B
	Nitrate nitrogen	2.81±0.29	2.76±0.31	3.13±0.59	3.16±0.28B
	CK	1.00±0.07	1.00±0.07	1.00±0.07B	1.00±0.07B
ON	Organic nitrogen	1.00±0.07c	1.10±0.01b	1.22±0.05aA	1.22±0.09aA
(mg/kg)	Ammonium nitrogen	1.00±0.07	1.04±0.08	1.13±0.13AB	1.16±0.08A
	Nitrate nitrogen	1.00±0.07	1.02±0.07	1.11±0.19AB	1.04±0.04B

Note: D10, D30, D60, and D90 represent days 10, 30, 60, and 90 of culture; Significance between nitrogen sources is indicated by "ABC" and between nitrogen application is indicated by "abc".

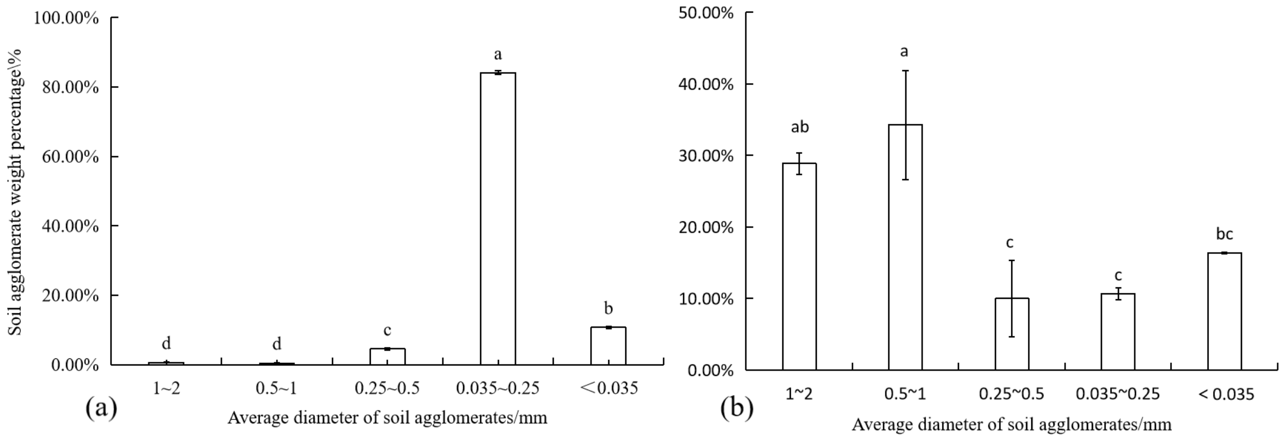

Figure. S2. Soil agglomerate particle size of temperate steppe (a) and warm-temperate shrub (b).

Table S3

Soil agglomerates MWD, GWD, and D of temperate steppe (a) and warm-temperate shrub (b).

	**MWD**	**GWD**	**D**
TS	0.1542b	0.1326b	2.0262a
WTS	0.7470a	0.4176a	1.5630b
