# Supplementary material for: Nitrogen-cycling microbial communities respond differently to nitrogen addition under two contrasting grassland soil types
Source: Front Microbiol. 2024 May 30;15:1290248. doi: 10.3389/fmicb.2024.1290248 (PMC11169941; doi:10.3389/fmicb.2024.1290248)
Supplement: Supplementary file 2 [file Presentation_2.pdf]

- N addition significantly affects soil N cycling microbial communities.
- The effect of N source on the N cycle microbial community was more significant.
- N addition causes the soil N cycle to be concentrated in the process of N fixation.
- Changes in environmental factors due to N deposition bring positive effects.
